# Pan-Cancer Analysis of PGAM1 and Its Experimental Validation in Uveal Melanoma Progression

**DOI:** 10.7150/jca.93398

**Published:** 2024-02-17

**Authors:** Weihong Niu, Yan Yang, Yuetai Teng, Na Zhang, Xu Li, Yinhui Qin

**Affiliations:** 1 Department of Pathology, Henan Key Laboratory for Digital Pathology Medicine, Henan Provincial People's Hospital, Zhengzhou University People's Hospital, Henan University People's Hospital, Zhengzhou 450003, Henan, China.; 2 Department of Pharmacy, Henan Provincial People's Hospital, Zhengzhou University People's Hospital, Henan University People's Hospital, Zhengzhou 450003, Henan, China.; 3 Microbiome Laboratory, Henan Provincial People's Hospital, Zhengzhou University People's Hospital, Henan University People's Hospital, Zhengzhou 450003, Henan, China.; 4 Institute of Chemistry Henan Academy of Sciences, No. 56 Hongzhuan Road, Jinshui District, Zhengzhou 450002, China.; 5 Shandong Academy of Chinese Medicine, Jinan 250014, China.; 6 Department of Pharmacy, Jinan Vocational College of Nursing, Jinan 250102, China.

**Keywords:** PGAM1, UVM, Immune infiltration, EMT, Apoptosis

## Abstract

Phosphoglycerate mutase 1 (PGAM1) is a key enzyme regulating cancer glycolysis. However, the expression and function of PGAM1 in uveal melanoma (UVM) are unknown and systematic analysis is lacking. This study performed a comprehensive analysis of PGAM1 expression across 33 cancer types in multiple public databases. Results demonstrated PGAM1 is aberrantly overexpressed in most tumors compared to normal tissues, and this overexpression is associated with poor prognosis, advanced tumor staging, and aggressive clinical phenotypes in multiple cancers including UVM, lung, breast and bladder carcinomas. In addition, PGAM1 expression positively correlated with infiltration levels of tumor-promoting immune cells including macrophages, NK cells, myeloid dendritic cells, etc. Further experiments showed that PGAM1 was overexpressed in UVM cell lines and tissues, and it was positively associated with a poor prognosis of UVM patients. And knockdown of PGAM1 inhibited migration/invasion and induced apoptosis in UVM cells, followed by decreased levels of PD-L1, Snail, and BCl-2 and increased levels of E-cadherin. Additionally, the correlation analysis and molecular docking results suggest that PGAM1 could interact with PD-L1, Snail and BCl-2. Thus, PGAM1 may promote UVM pathogenesis via modulating immune checkpoint signaling, EMT and apoptosis. Collectively, this study reveals PGAM1 as a valuable prognostic biomarker and potential therapeutic target in aggressive cancers including UVM.

## Introduction

Cancer is a major global health challenge and the leading cause of mortality worldwide, resulting in approximately 10 million deaths in 2020 alone 1, 2. Among the various types of cancer, UVM is a rare but aggressive form of intraocular malignancy that originates from melanocytes within the uveal tract, comprising the iris, ciliary body, and choroid 3. UVM accounts for approximately 5% of all melanomas and has a high propensity to metastasize, especially to the liver 4. The 10-year survival rate of UVM patients is only about 50%, and the median survival after metastasis detection is less than 12 months 5-7. Despite advances in early diagnosis and therapeutic approaches, the prognosis of UVM remains dismal, largely due to the lack of effective treatments for metastatic disease and the poor understanding of the molecular mechanisms underlying UVM pathogenesis. Therefore, there is an urgent need to identify novel prognostic biomarkers and therapeutic targets for UVM. In this study, we investigated the role of PGAM1, a key glycolytic enzyme, in UVM progression and its potential as a therapeutic target.

Aberrant metabolic reprogramming, characterized by increased glycolysis and reduced oxidative phosphorylation, is a hallmark of cancer that supports its malignant properties, such as unrestrained proliferation, invasion, and therapy resistance 8, 9. Among the key enzymes involved in glycolysis, PGAM1 catalyzes the reversible conversion of 3-phosphoglycerate (3PG) to 2-phosphoglycerate (2PG), which is essential for maintaining the glycolytic flux and producing biosynthetic precursors. PGAM1 constitutes a key glycolytic enzyme that catalyzes the interconversion between 3PG and 2PG during later phases of glycolysis and gluconeogenesis 10. Accumulating studies have revealed that PGAM1 is frequently overexpressed in various human cancers and correlates with poor prognosis and increased metastasis, including colon, breast, lung, and hepatocellular carcinoma 11-17. Therefore, elucidating the role of PGAM1 in tumor metabolism and other biological processes could contribute to evaluating its potential as a therapeutic target for cancer treatment. Previous studies have revealed that PGAM1 has a metabolic function in promoting homologous recombination (HR) repair, which facilitates DNA double-strand break (DSB) end-resection by regulating the stability of the C-terminal binding protein interacting protein (CtIP) 18. Besides its metabolic role, PGAM1 can also interact with α-smooth muscle actin (ACTA2) to enhance tumor growth and metastasis and overcome erlotinib resistance in non-small-cell lung cancer (NSCLC) 19. Moreover, PGAM1 can promote prostate cancer (PCa) angiogenesis and metastasis by binding to γ-actin (ACTG1) 20. Additionally, elevated PGAM1 expression correlates with clinical features such as tumor grade, lymph node metastasis, and pathological staging, serving as an independent risk factor affecting prognosis. PGAM1-mediated glycolytic metabolism plays an important role in paclitaxel resistance, and overexpression of PGAM1 increased glycolytic flux and paclitaxel resistance in SKOV3 paclitaxel-sensitive ovarian cancer cells 21. However, the expression patterns, clinical significance, and biological functions of PGAM1 in UVM have been scarcely investigated.

In this study, the mRNA and protein expression patterns, genetic variation, and prognostic correlation of PGAM1 in UVM were analyzed by bioinformatics methods. The effects of PGAM1 on migration/invasion and apoptosis of UVM cells, and its molecular mechanism were investigated by biochemical and molecular biology techniques. The overexpression of PGAM1 in UVM tumor tissues was verified by immunohistochemistry. In addition, this study also used biological information to predict the upstream miRNA and downstream signaling pathways bound by PGAM1, applied molecular docking techniques to predict the binding patterns of PGAM1 and other key effector molecules, constructed upstream and downstream regulatory networks related to PGAM1, and clarified its core role in the development of UVM. As a key enzyme of glycolysis, the abnormal activation of PGAM1 is closely related to the occurrence and development of various malignant tumors. However, its role in the pathogenesis of UVM has received little attention. This study systematically evaluates the role of PGAM1 in the development of UVM and constructs a regulatory network with PGAM1 as the core signal hub. We found that high expression of PGAM1 predicted poor prognosis of UVM patients and promoted migration/invasion and immune escape of UVM cells. In addition, PGAM1 regulates apoptosis, epithelial mesenchymal transformation, and tumor immune microenvironment by up-regulating effector molecules such as Bcl2, Snail, and PD-L1. Therefore, PGAM1 may be a key driver of UVM progression and a promising therapeutic target and prognostic biomarker. This study provides a new perspective for individualized diagnosis and treatment of UVM.

## Methods

### Data retrieval and processing

The RNA sequencing data of PGAM1 expression and matched clinical information across 33 cancer types were retrieved from TCGA database (https://portal.gdc.cancer.gov/) and GTEx portal (https://www.gtexportal.org/). The on-chip sequencing data (Affymetrix Human Genome U133 Plus2.0 Array platform) and the clinical information files, designated GSE22138 22, were also collected from the GEO database (https://www.ncbi.nlm.nih.gov/geo/) download. The cancer types included were ACC, Adrenocortical carcinoma; BLCA, Bladder Urothelial Carcinoma; BRCA, Breast invasive carcinoma; CESC, Cervical squamous cell carcinoma and endocervical adeno carcinoma; CHOL, Cholangio carcinoma; COAD, Colon adenocarcinoma; DLBC, Lymphoid Neoplasm Diffuse Large B-cell Lymphoma; ESCA, Esophageal carcinoma; GBM, Glioblastoma multiforme; HNSC, Head and Neck squamous cell carcinoma; KICH, Kidney Chromophobe; KIRC, Kidney renal clear cell carcinoma; KIRP, Kidney renal papillary cell carcinoma; LAML, Acute Myeloid Leukemia; LGG, Brain Lower Grade Glioma; LIHC, Liver hepatocellular carcinoma; LUAD, Lung adenocarcinoma; LUSC, Lung squamous cell carcinoma; MESO, Mesothelioma; OV, Ovarian serous cystadenocarcinoma; PAAD, Pancreatic adenocarcinoma; PCPG, Pheochromocytoma and Paraganglioma; PRAD, Prostate adenocarcinoma; READ, Rectum adenocarcinoma; SARC, Sarcoma; SKCM, Skin Cutaneous Melanoma; STAD, Stomach adenocarcinoma; TGCT, Testicular Germ Cell Tumors; THCA, Thyroid carcinoma; THYM, Thymoma; UCEC, Uterine Corpus Endometrial Carcinoma; UCS, Uterine Carcinosarcoma; UVM, Uveal Melanoma. Immunohistochemistry images of PGAM1 protein expression across normal and cancerous tissues were downloaded from the Human Protein Atlas database Human Protein Atlas Information (HPA, https://www.proteinatlas.org/) 23.

### Prognostic Implications of PGAM1 Expression in Human Malignancies

Prognostic data based on gene expression were obtained from the TCGA and UCSC Xena database, involving four outcome types: overall survival (OS), disease-specific survival (DSS), disease-free interval (DFI), and progression-free interval (PFI). The impact of PGAM1 expression on cancer-specific prognosis for each outcome type was evaluated using univariate Cox regression and Kaplan-Meier models. A heatmap showed the prognostic results for each cancer type, including log-rank p-values calculated using the Kaplan-Meier method and hazard ratios (HR) with 95% confidence intervals (95% CI). In addition, we obtained high-quality TCGA prognostic data set from the previous TCGA prognostic study published in Cell and eliminated the cancer species with less than 10 samples in a single cancer species 24, 25. We performed log2 transformations on the TPM values in the RNA sequencing data to reduce the skewness and variance of the data. We used R software (version 4.2.1), ggplot2 package (3.3.6) and timeROC package (version 0.4) for AUC analysis.

### Tumor mutation burden (TMB) and microsatellite instability (MSI)

RNA sequencing data and corresponding clinical information for tumors were obtained from the Cancer Genome Atlas (TCGA) database (https://portal.gdc.com). TMB was sourced from the article "The Immune Landscape of Cancer" published by Vestian Thorsson et al. in 2018 26; MSI was sourced from the article "Landscape of Microsatellite Instability Across 39 Cancer Types" published by Russell Bonneville et al. in 2017^26^.

### Gene Set Enrichment Analysis of PGAM1 in Cancers

The biological functions and potential signaling pathways of PGAM1 in different tumors were investigated by us using Gene set enrichment analysis (GSEA, version 4.3.2) software and MsigDB database (version 7.0), using the pre-defined Canonical Pathways and Hallmarks gene sets from the Molecular Signatures Database (MSigDB). Significant terms were defined as p < 0.05. GRAMM: Docking Web Server (http://gramm.compbio.ku.edu/) 28-30 was used to perform docking analysis of PGAM1 and its related gens.

### Target miRNA Prediction and ceRNA Network Construction

Targets of up- and down-regulated miRNAs that affect PGAM1 expression were predicted using three databases, namely DIANA-microT (http://diana.imis.athena-innovation.gr/DianaToolsindex.php?r=microTCDS/index) and miRDB (http://mirdb.org/miRDB/) 31, 32. Then, miRNA targets, lncRNA, and circRNA information related to PGAM1 were determined using RNA-RNA interactome in ENCORI platform (https://rnasysu.com/encori/index.php) 33. Finally, the lncRNA-miRNA-mRNA ceRNA network was visualized using Cytoscape 3.10.1.

### Immune infiltration analysis

The Tumor Immune Estimation Resource (TIMER) is an important data resource that enables the quantification of immune infiltrates in different cancer types. We obtained the immune cell infiltration level data for TCGA cancers from the TIMER2 database. Based on this pan-cancer resource, we investigated the correlation between PGAM1 mRNA expression and 11 immune cell subgroups, which include B lymphocytes, CD8+ T lymphocytes, CD4+ T lymphocytes, regulatory T cells, macrophages, natural killer cells, myeloid dendritic cells, monocytes, cancer-associated fibroblasts, mast cells, and neutrophils.

Using the online website Sangerbox (http://sangerbox.com/home.html) 25, the expression level of PGAM1 was correlated with immune score, ESTIMATE score and stromal score based on tumor samples from the TCGA database. Each point represents a sample, and the color represents different cancer types. The correlation coefficient and p-value were calculated by the Spearman method. The heatmap was drawn, and the color represents the magnitude of the correlation coefficient, red for positive correlation and blue for negative correlation.

### Cell proliferation

To assess the growth potential of transfected cells, we utilized the Cell Counting Kit-8 (CCK-8) assay from New Cell & Molecular Biotech Co., Ltd, China. The method involved seeding transfected cells at a density of 1×10^3 cells per well into a 96-well plate, followed by overnight incubation. Subsequently, at time points 0, 1, 2, 3 and 4 days, we added 10 µL of CCK-8 solution to each well and recorded the absorbance at a wavelength of 450 nm.

### Scratch wound healing assay

A wound healing assay was conducted to assess the migratory ability of C918 cells. C918 cells were seeded in 6-well plates and cultured to confluence. At 95% confluence, a scratch wound was generated in each well using a 10 μL micropipette tip. Cells were rinsed with PBS and incubated in low serum medium. Phase contrast images of the wound area were captured at regular intervals. The scratch wound healing rate was quantified by measuring the wound width at different time points compared to the initial wound width at 0 h using Image J software.

### Transwell invasion assay

A transwell invasion assay was conducted to evaluate cell migratory ability. In brief, cells were resuspended in 200 μL of serum-free medium and seeded into the upper chambers of 24-well inserts containing 8 μm porous membranes (Corning, NY, USA). The lower chambers were filled with medium supplemented with 20% fetal bovine serum (FBS) as a chemoattractant. Following incubation, non-migrated cells on the upper membrane surface were removed by cotton swab scraping. In contrast, successfully transmigrated cells on the lower surface were fixed with 4% polyformaldehyde for 30 minutes and subsequently stained with 0.1% crystal violet solution. Five random fields per insert were imaged under light microscopy. The number of invaded cells per field were quantified by digital image analysis using ImageJ software.

### Western blotting

Cell lysates were prepared using WB and IP lysis buffers (New Cell & Molecular Biotech Co., Ltd, China) and protein concentrations were measured by BCA assay (New Cell & Molecular Biotech Co., Ltd, China). Servicebio Prestained Protein Marker II (10-200 kDa) (Servicebio, #G205B) was used as a protein marker to monitor protein migration and transfer. Equal amounts of protein (60 μg) were loaded onto 10% SDS-PAGE gels and separated by electrophoresis. Then, the proteins were transferred to 0.22 μM PVDF membranes (Millipore, Billerica, MA, USA) using a semi-dry transfer system. The membranes were incubated with 5% non-fat milk for 1 h at room temperature to block non-specific binding. Next, the membranes were probed separately overnight at 4 °C with primary antibodies against PGAM1 (Proteintech, 1:1000), c-PARP (Abways, 1:1000), E-cadherin (Proteintech, 1:1000), Bcl-2 (Proteintech, 1:1000), Snail (Proteintech, 1:1000), PD-L1 (Abcam, 1:1000), and GAPDH (Abways, 1:3000) as a loading control. Following 1*PBST washes, membranes were incubated with appropriate HRP-conjugated secondary antibodies for 1 h at 37 °C. HRP-conjugated Affinipure Goat Anti-Mouse IgG (H + L) (Proteintech, 1:5000 dilution) and HRP-conjugated affinity purified goat anti-rabbit IgG (H+L) (Proteintech, 1:5000 dilution) were used as the secondary antibodies in Western blot. Immunoreactive bands were visualized using ECL substrate (Abbkine, China) after final PBST washes.

### TUNEL assay

Fluorescein (FITC) Tunel Cell Apoptosis Detection Kit was purchased from (G1501, Servicebio, Wuhan, China) was performed to detect nuclear DNA breaks in C918 cells during late apoptosis according to the manufacturer's instructions. Images were acquired using an Olympus fluorescent microscope (Olympus Corporation, Tokyo, Japan). FITC-12-dUTP was incorporated into apoptotic nuclei and localized as green fluorescence.

### Patients and Specimens

From May 2020 to May 2023, 56 pairs of paraffin-embedded specimens of uveal melanoma and corresponding adjacent non-tumor tissues were obtained in the separtment of Pathology at Henan Provincial People's Hospital. Ethical approval was obtained from the the Medical Ethics Committee of Henan Provincial People's Hospital.

### Statistics Analysis

Statistical analyses were conducted utilizing R 4.2.1, SPSS 23.0 and GraphPad Prism 9.5 software. Differences between two experimental groups were evaluated via two-tailed Student's t-tests. One-way ANOVA with post-hoc testing was performed for comparisons of three or more groups, with p-values adjusted for multiple comparisons. Categorical data were analyzed using Chi-square tests. Quantitative data are presented as mean ± standard deviation. The p values are labeled in figures and were denoted as follows: *p < 0.05, **p < 0.01, ***p <0.001 and ****p <0.0001.

## Results

### PGAM1 is aberrantly dysregulated across diversified human cancers

**Figure 1:**
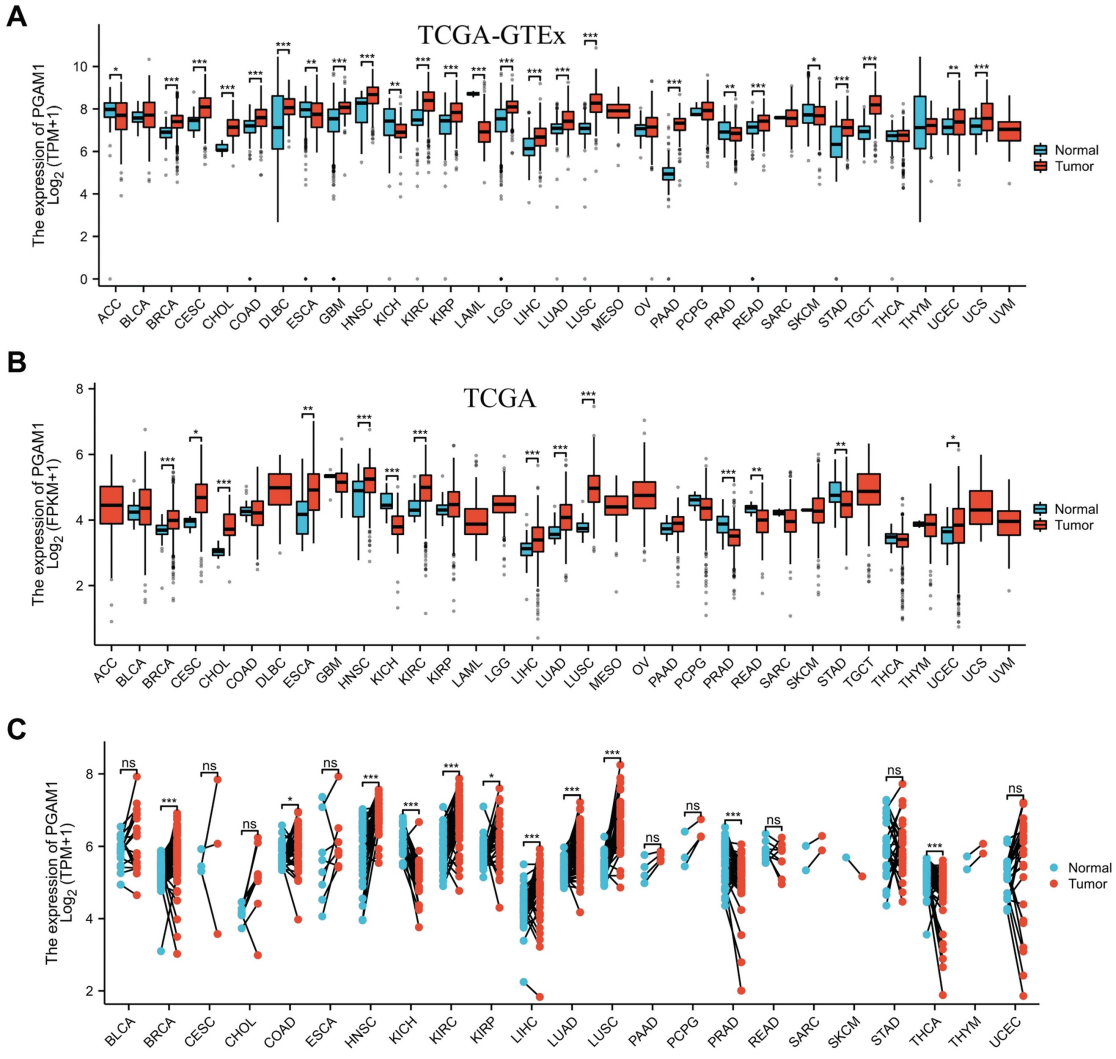
PGAM1 expression analysis in pan-cancer. **(A)** Analysis of PGAM1 expression levels between 33 different tumor types and normal tissues based on combined TCGA and GTEx data bases.** (B)** Analysis of PGAM1 expression in tumor versus normal samples across 18 cancer types using TCGA database. **(C)** Paired analysis of PGAM1 expression in tumor tissues compared to matched normal controls across 18 tumor types based on TCGA data. ∗p < 0.05, ∗∗p < 0.01, ∗∗∗p < 0.001.

In order to elucidate the expression pattern of PGAM1 in pan-cancer, we analyzed the TCGA_GTEx datasets of 33 tumors collected from TCGA-GTEx. The results showed that PGAM1 was differentially expressed in most tumor tissues, with upregulation in most tumors while downregulation in a few (**Figure 1A**). This is largely consistent with the results from the TCGA database (**Figure 1B**). In addition, our analysis of 23 pairs of matched tumor and non-tumor tissues from TCGA revealed that PGAM1 is typically overexpressed in the tumor tissues (**Figure 1C**). The TNM staging results indicated that PGAM1 was upregulated in high-stage tumor tissues (**Supplementary Figure 1**). Besides, the expression of PGAM1 protein in normal and tumor tissues from various organs was investigated by using the Human Protein Atlas (HPA) database. Representative immunohistochemistry (IHC) images were chosen to demonstrate the results (**Supplementary Figure 2A**). Additionally, the survival analysis revealed that high PGAM1 expression correlated with poor prognosis compared to low expression (**Supplementary Figure 2B**). Moreover, the CPTAC database analysis showed that PGAM1 protein was significantly upregulated in Lung, LUSC, PAAD, and ccRCC tissues relative to normal tissues (**Supplementary Figure 2C**).

### Aberrant DNA Methylation Patterns of PGAM1 Across Cancers

**Figure 2:**
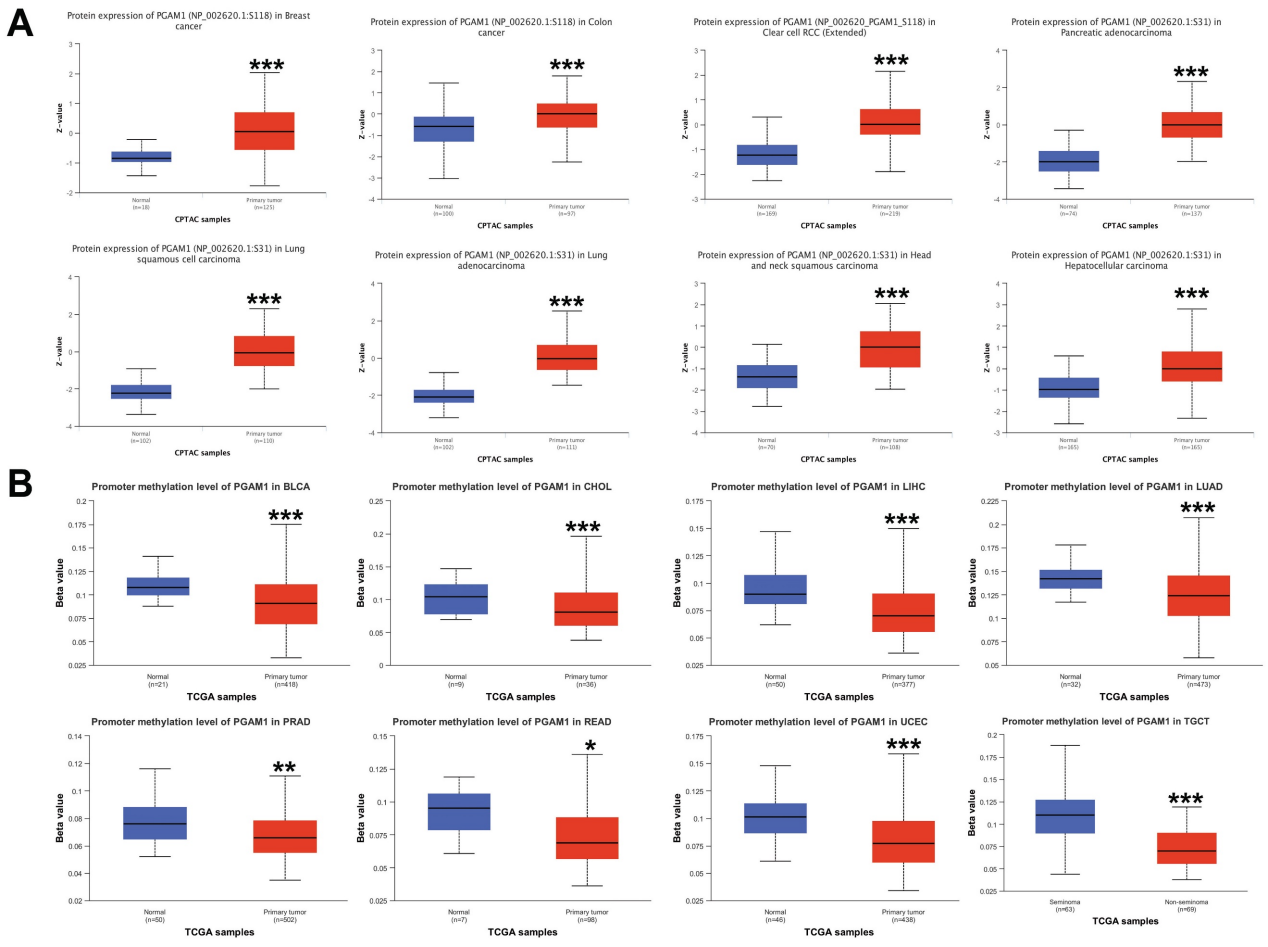
Pan-cancer PGAM1 protein phosphorylation and DNA methylation. **(A)** CPTAC shows the phosphorylation levels of PGAM1 at S118 or S31 sites. **(B)** PGAM1 DNA methylation in normal and primary tumor tissues based on UALCAN database: blue represents healthy control group; red represents tumor patients.

The differences in the phosphorylation levels of PGAM1 in normal and primary tumor tissues were further explored. Using the CPTAC dataset, we found that phosphorylation of the PGAM1 S118 site was higher in cancers such as BRCA, COAD, and ccRCC compared to normal tissues, while phosphorylation of the PGAM1 S31 site was higher in cancers such as PAAD, LUSC, LUAD, HNSC and HCC compared to normal tissues (**Figure 2A**). DNA methylation is an important epigenetic modification that can regulate the expression of cancer-related genes. However, the mechanistic role of PGAM1 methylation in cancer has been unclear. We found that PGAM1 methylation levels were significantly lower in cancers such as BLCA, CHOL, LIHC, LUAD, PRAD, READ, UCEC and TGCT compared to normal tissues (**Figure 2B**). This implies that hypomethylation of PGAM1 may promote the development of various cancers. In addition, we analyzed the correlation between PGAM1 and methylation-related genes, and the results showed that PGAM1 had significant correlations with methyltransferases such as DNMT1, DNMT3A and DNMT3B as well as methylation modifications such as m1A, m5C and m6A across various cancers (**Supplementary Figure 3**).

### PGAM1 associates with clinical outcomes in multiple aggressive cancers

**Figure 3:**
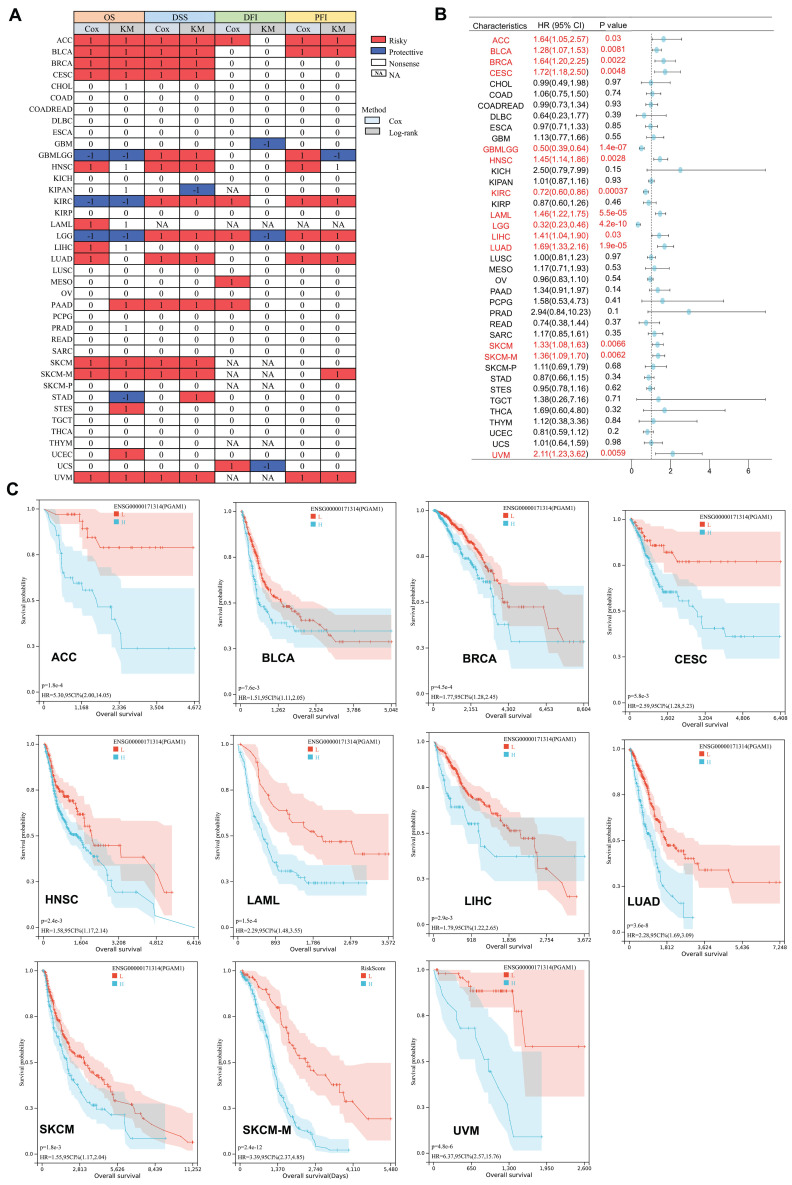
The Prognostic Significance of PGAM1. **(A)** The relationship between PGAM1 expression and patient outcomes, including OS, DSS, DFI, and PFI, was assessed using both univariate Cox regression and Kaplan-Meier analysis. PGAM1 is indicated in red as a risk factor impacting the prognosis of cancer patients, whereas blue denotes a protective effect. Only p-values < 0.05 are displayed. **(B)** Univariate Cox regression analysis was conducted to evaluate the role of PGAM1 in pan-cancer survival (OS). **(C)** Kaplan-Meier survival curves for PGAM1 in diverse cancer types, including ACC, BLCA, BRCA, CESC, HNSC, LAML, LIHC, LUAD, SKCM, SKCM-M and UVM.

Previous studies have shown high expression of PGAM1 in multiple tumor types. To further investigate the prognostic value of PGAM1 in cancers, OS prognostic heatmap analysis was performed. The results demonstrated that PGAM1 was associated with poor OS and was identified as a risk factor. Additionally, DSS, DFI, and PFI were assessed to evaluate the effects of PGAM1 on prognosis of various cancers. PGAM1 was determined to be a risk factor in most cancer types based on these analyses (**Figure 3A**). Cox regression analysis of OS revealed that high expression of PGAM1 correlated with poor prognosis in ACC (HR=1.64[95%CI,1.05-2.57], BLCA (HR=1.28[95%CI,1.07-1.53], BRCA (HR=1.64[95%CI,1.20-2.25], CESC (HR=1.72[95%CI,1.18-2.50], HNSC (HR=1.45[95%CI,1.14-1.86], LAML (HR=1.46[95%CI,1.22-1.75], LIHC (HR=1.41[95%CI,1.04-1.90], LUAD (HR=1.69[95%CI,1.33-2.16], SKCM (HR=1.33[95%CI,1.08-1.63], SKCM-M (HR=1.36[95%CI,1.09-1.70] and UVM (HR =2.11[95%CI,1.23-3.62]) patients, while it was a protective factor for GBMLGG (HR=0.50[95%CI,0.39-0.64], KIRC (HR=0.72[95%CI,0.60-0.86]) and LGG (HR=0.32[95%CI,0.23-0.46]) patients (**Figure 3B**).

Furthermore, Kaplan-Meier curves analysis further revealed that lower PGAM1 expression correlated with improved survival outcomes in ACC, BLCA, BRCA, CESC, HNSC, LAML, LIHC, LUAD, SKCM, SKCM-M and UVM (**Figure 3C**), suggesting PGAM1 was a poor prognostic biomarker for these cancers. At the same time, we collected and analyzed the clinical data of ACC, BLCA, BRCA, CESC, HNSC, LAML, LIHC, LUAD, SKCM and UVM patients. The patients were divided into high expression group and low expression group according to the expression level of PGAM1. The results showed that the pathological stage and clinical M stage were significantly correlated with the expression of PGAM1 in ACC (**Supplementary Table 1**). In CESC, the expression of PGAM1 was significantly correlated with pathological T-stage, clinical stage and pathological type (**Supplementary Table 2**). In UVM, the expression of PGAM1 was significantly correlated with pathological stage, clinical stage and clinical T stage (**Supplementary Table 3**). In LUAD, the expression of PGAM1 was significantly correlated with pathological T, N, M stage and pathological stage (**Supplementary Table 4**). In HNSC and SKCM, radiotherapy is significantly associated with PGAM1 expression (**Supplementary Table 5-6**). In BLCA, primary therapy outcome is significantly associated with PGAM1 expression (**Supplementary Table 7**). Additionally, a ROC curve was used to evaluate the diagnostic value of PGAM1, and it was found that tumors with PGAM1 ratio (AUC) greater than 0.6 under ROC curve were ACC (AUC=0.845), CESC (AUC=0.648), LAML (AUC=0.627), LIHC (AUC=0.676), MESO (AUC=0.611), PAAD (AUC=0.618), SARC (AUC=0.605), TGCT (AUC=0.833) and UVM (AUC=0.734), respectively (**Supplementary Figure 4**).

### PGAM1 Single-Cell Analysis in Cancer

A heatmap depicting PGAM1 expression levels of 39 datasets, including immune cells, stromal cells, malignant cells, and functional cells, using the TISCH web tool. The results indicate prominent expression of PGAM1 in both immune cells, especially tumor-associated macrophages, and in malignant cells (**Supplementary Figure 5A**). In the glioblastoma dataset GSE102130, we analyzed single-cell transcriptomic profiles of 3321 cells from 6 glioblastoma patients. We found that protein PGAM1 was highly expressed in both malignant glioblastoma cells as well as tumor-associated monocyte/macrophage cells within the glioblastoma microenvironment (**Supplementary Figure 5B**). In the GSE139829 dataset, which contains 103703 cells from 8 primary and 3 metastatic samples UVM patients, PGAM1 expression is widely expressed across immune cell types like T cells, monocytes, or macrophages in the UVM microenvironment (**Supplementary Figure 5C**).

### Analysis of the correlation between PGAM1 and immune cell infiltration

**Figure 4:**
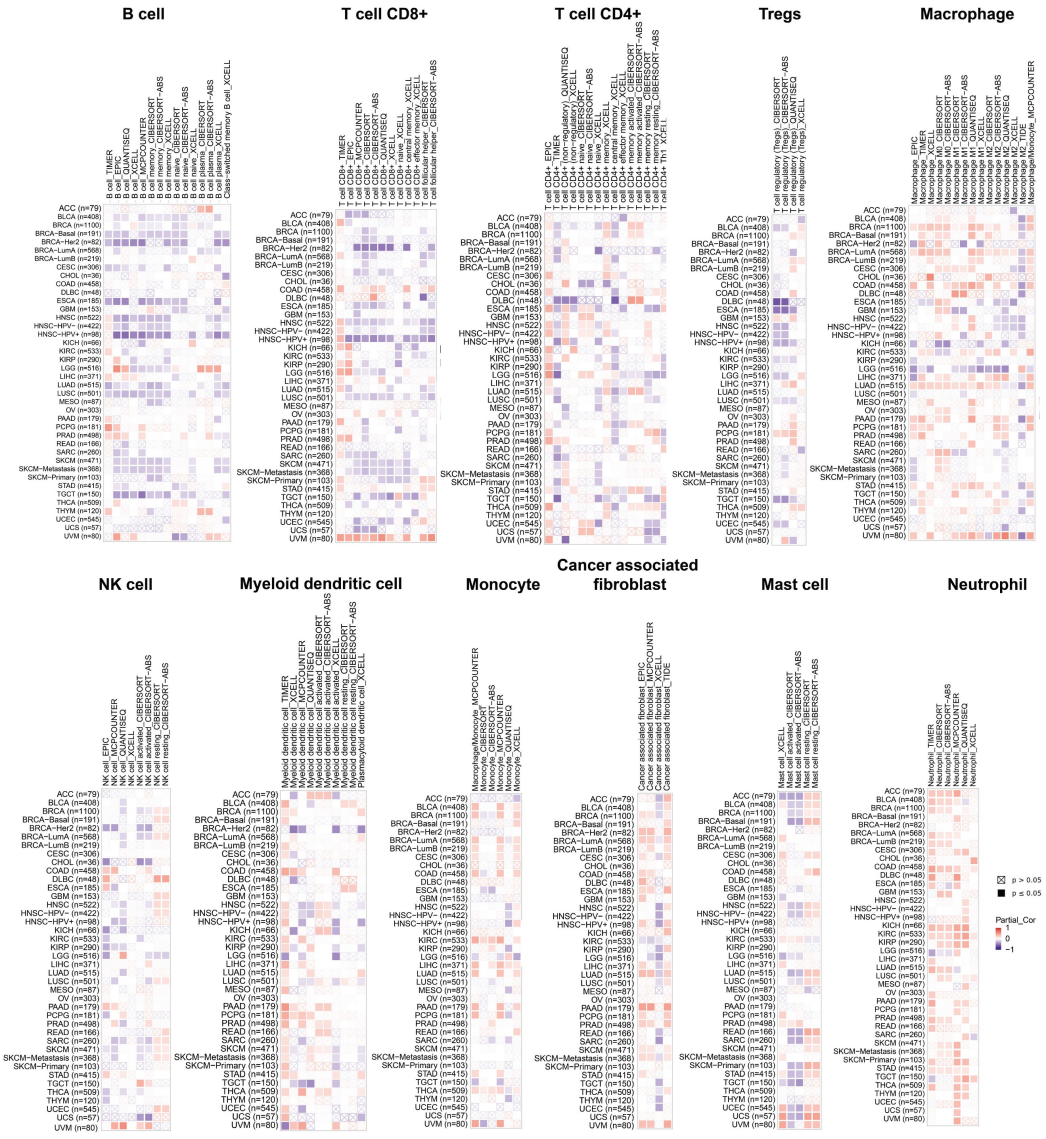
Relationship analysis of PGAM1 and immune cell infiltration. The relationship between PGAM1 expression and the infiltration levels of B cells, CD8+ T cells, CD4+ T cells, Tregs, Macrophages, NK cells, Myeloid dendritic cells, Monocytes, Cancer-associated fibroblasts, Mast cells and Neutrophils. Positive correlation is indicated in red font and negative correlation in blue font.

Recent studies have demonstrated that infiltration of immune cells such as CD4+ T cells, CD8+ T cells, myeloid-derived suppressor cells and cancer-associated fibroblasts plays a critical role in cancer immunotherapy 34, 35. The relationship between PGAM1 expression nd immune cell infiltration was investigated to demonstrate the link between PGAM1 and cancer immunity. Spearman correlation analysis was conducted utilizing the pooled pan-cancer immune cell infiltration data from the TIMER2 database. The results showed that in most TCGA cancers, PGAM1 expression positively correlated with infiltration levels of macrophages, natural killer cells, myeloid dendritic cells, monocytes, cancer-associated fibroblasts, mast cells and neutrophils. Additionally, PGAM1 expression positively correlated with infiltration levels of numerous immune cells including CD4+ T cells, CD8+ T cells, regulatory T cells, macrophages, natural killer cells, myeloid dendritic cells, monocytes and cancer-associated fibroblasts in UVM (**Figure 4**). We comprehensively analyzed the correlation of PGAM1 with ImmuneScore, EstimateScore and StromalScore in in multiple tumors. The results demonstrated positive correlations between PGAM1 expression and the ImmuneScore, EstimateScore, and StromalScore in LAML, KIPAN, PAAD, DLBC, UVM, READ, PCPG, COADREAD, PRAD, KIRC, LUAD and BLCA. Conversely, negative correlations were observed between PGAM1 expression and these scores in HNSC, LUSC, THYM, SKCM, ESCA, SARC, STES, ACC, GBMLGG, TGCT, LGG and UCEC (**Supplementary Figure 6**). In summary, our findings suggest PGAM1 may regulate cancer progression, prognosis, and treatment response by modulating immune cells in the tumor microenvironment.

Correlation analysis shows PGAM1 expression correlates with infiltration levels of various immune cells including macrophages, natural killer cells, myeloid dendritic cells, monocytes, cancer-associated fibroblasts, mast cells, neutrophils, B cells, CD4+ T cells, CD8+ T cells and regulatory T cells. These immune cells are involved in anti-tumor immunity and sometimes cancer promotion. Thus, PGAM1 interactions with immune cells likely influence the immunologic landscape of the tumor niche, impacting cancer development and therapy response.

### Correlation analysis between PGAM1 and immune regulatory genes, TMB and MSI

**Figure 5:**
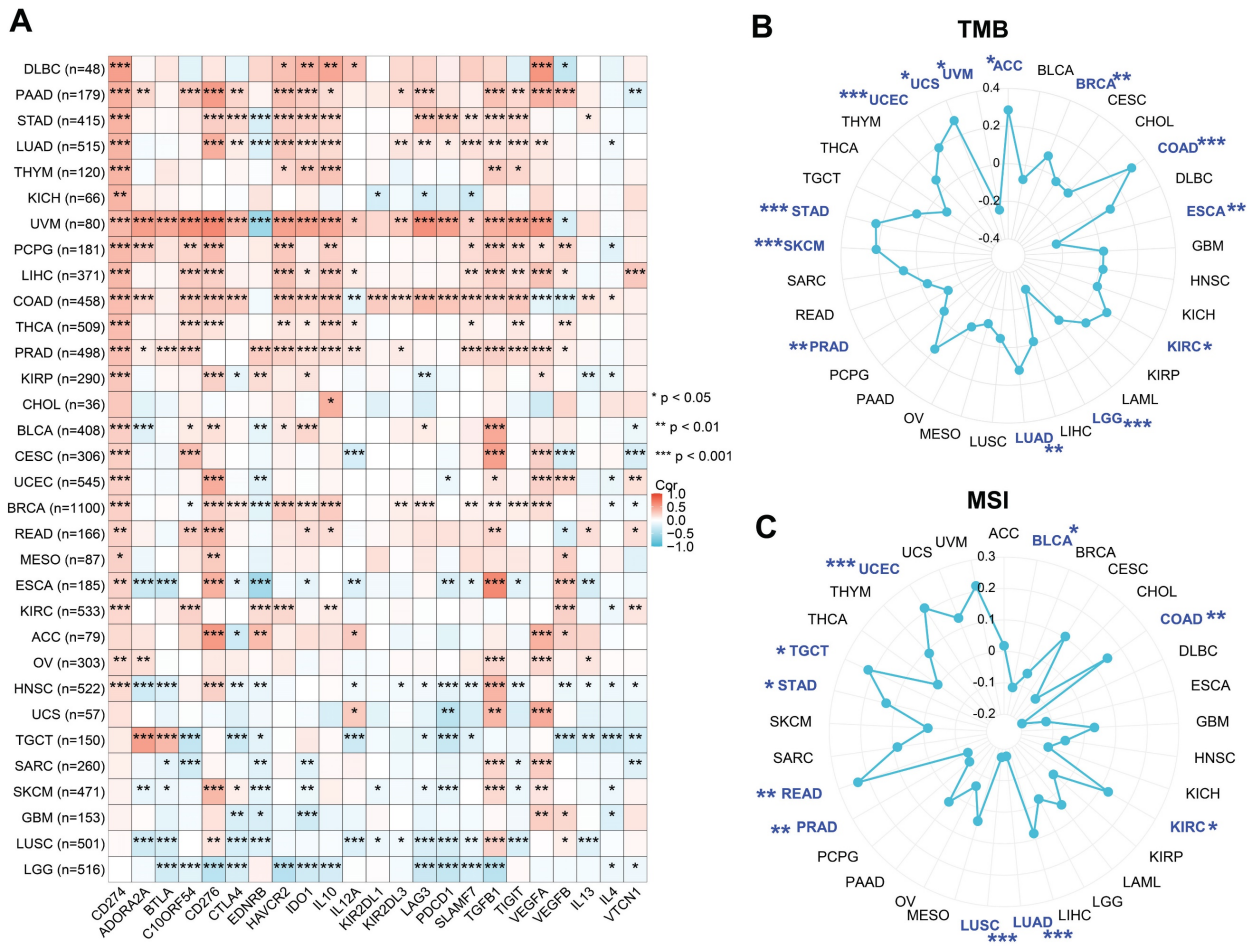
The correlations between PGAM1 expression and various ICGs, TMB and MSI across diverse cancer types. **(A)** Heatmaps show the correlation coefficients between PGAM1 and ICGs in different cancer types. The color scale ranges from blue (negative correlation) to red (positive correlation). **(B)** A radar chart displays the correlation coefficients between PGAM1 expression and TMB in different cancer types. **(C)** A radar chart displays the correlation coefficients between PGAM1 expression and MSI in different cancer types. The blue regions indicate significant correlation (p < 0.05).

The correlation of PGAM1 with 23 immune checkpoint genes (ICGs) in different cancer types was shown using **Figure 5A**. It was found that PGAM1 expression was associated with various ICGs in different cancer types, but the direction and strength of the association varied. For instance, PGAM1 expression was strongly positively correlated with ICGs (especially CD274) in UVM tumors, while it was significantly negatively correlated with most ICGs in LGG and LUSC tumors. The relationship of PGAM1 expression with TMB and MSI was further analyzed to evaluate whether PGAM1 expression could serve as a predictive factor for the efficacy of immune checkpoint inhibitors (ICIs). TMB 36 and MSI 37 were two novel biomarkers that could predict the response to ICIs. All tumor types were selected from the TCGA dataset and the correlation coefficients of PGAM1 expression with TMB and MSI were calculated. The results showed that PGAM1 expression was correlated with TMB in ACC, UVM, UCS, UCEC, STAD, SKCM, PRAD, LUAD, LGG, KIRC, ESCA, COAD and BRCA (**Figure 5B**). Moreover, PGAM1 expression was correlated with MSI in UCEC, TGCT, STAD, READ, PRAD, LUSC, LUAD, KIRC, COAD and BLCA (**Figure 5C**). We examined the correlation between PGAM1 and immunotherapy response through the ICBatlas website 38 (http://bioinfo.life.hust.edu.cn/ICBatlas/) and Tiger website (http://tiger.canceromics.org/#/home). Patients with high PGAM1 expression in the SRP183455/SRP217040, ERP107734, SRP217040, PRJEB25780, PRJEB23709 immunotherapy cohorts had significant immunotherapy responses, whereas patients with low PGAM1 expression in the SRP150548 cohort had poor symptomatic immunotherapy response rates (**Table 1** and **Supplementary Figure 7**). These results suggested that high PGAM1 expression might be related to the immune response in some tumors, but the underlying mechanisms needed to be further investigated.

### Prediction of Target miRNAs and Construction of the Co-Expressed Network

To investigate the role of miRNAs in gene regulation, we first examined the interactions between mRNAs, miRNAs and their corresponding ncRNAs, which form the ceRNA network. MiRNAs can bind to mRNAs and induce gene silencing or downregulation of gene expression. NcRNAs, such as circRNAs and lncRNAs, can act as upstream molecules and bind to miRNA response elements, thereby influencing the function of miRNAs and upregulating gene expression 39. We searched two databases and identified two miRNAs (miR-92b-3p and miR-17-3p) that target PGAM1, a key enzyme in glycolysis. We then used StarBase to predict 23 lncRNAs and 32 circRNAs that interact with these miRNAs, among which MALAT1 and XIST were lncRNAs that were common targets of miR-92b-3p and miR-17-3p. As shown in **Supplementary Figure 8**, our ceRNA network analysis results were consistent with the predictions, which can support our study.

### PGAM1 Regulates the Progression of UVM Through EMT, apoptosis and Immune Response Pathway

**Figure 6:**
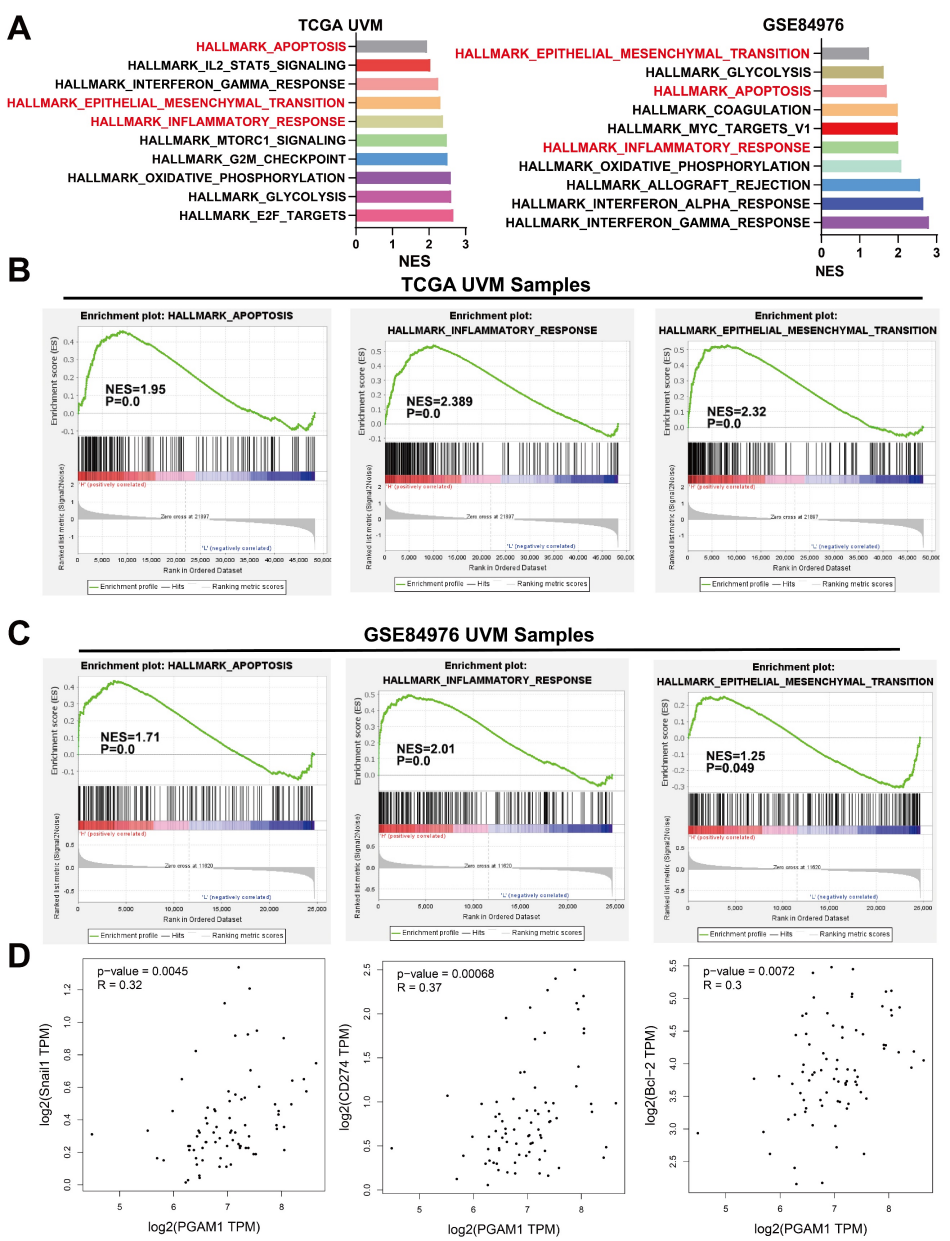
PGAM1 Expression is Associated with Immune Response and Apoptosis. **(A-C)** GSEA functional enrichment analysis of PGAM1 expression bases on TCGA and GSE84976 dataset. **(D)** PGAM1 expression positively correlated with genes involved in immune response, apoptosis and EMT pathway.

**Figure 7:**
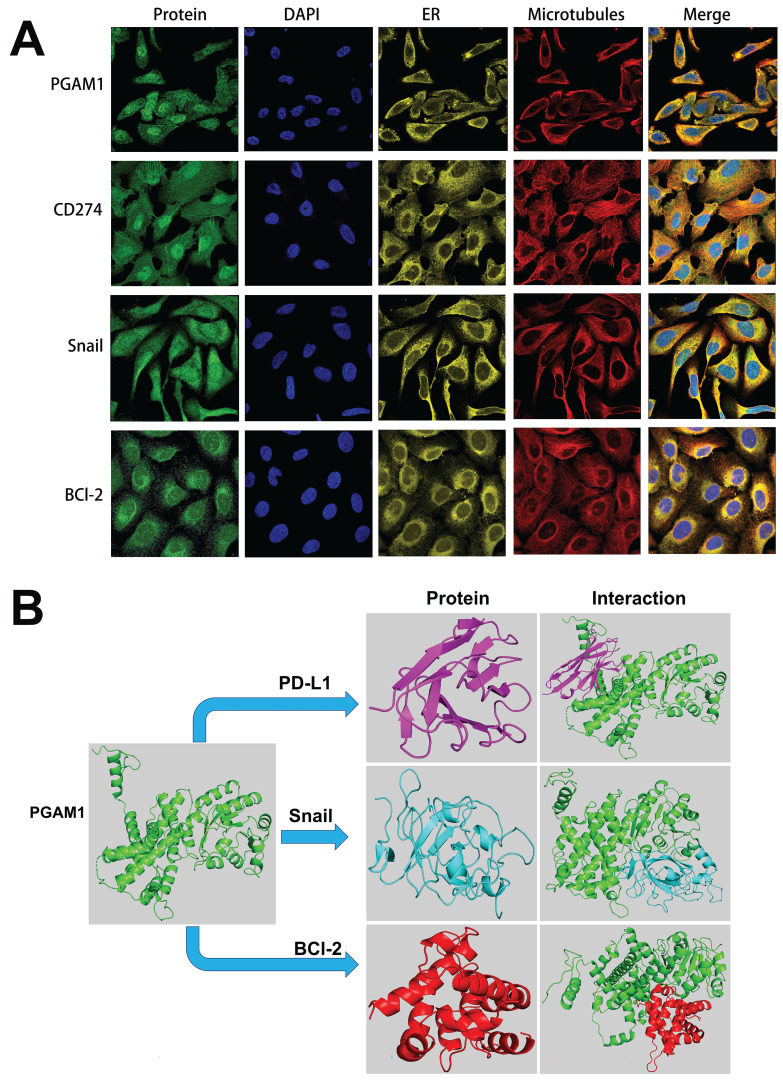
Cellular localization and molecular docking of PGAM1 interacting genes. **(A)** Cellular location of proteins PGAM1, CD274, Snail, bcl-2: green is the target protein, red is microtubule, yellow is endoplasmic reticulum, blue is cell nucleus (scale bar, 20μm). **(B)** The protein docking results of PGAM1 (PDB: 4gwg) with PD-L1 (PDB: 5dxw), BCl2 (PDB: 5tzp) and BCl2 (PDB: 5tzp), respectively. Green shows the PGAM1 protein structure. Purple is the PD-L1 protein structure, blue is the snail protein structure, and red is the BCl-2 protein structure. PD-L1 (PDB: 5dxw) interacted with PGAM1 (PDB: 4gwg) and BCl2 (PDB: 5tzp).

To elucidate the mechanistic involvement of PGAM1 in UVM pathogenesis, we performed GSEA enrichment analyses in both TCGA and GEO datasets, which concurrently revealed enrichment of EMT, apoptosis, inflammatory response and pathways in the PGAM1 high expression group (**Figure 6A-6C**). Recent studies have discovered that immune responses and the tumor immune microenvironment modulate tumor progression 40. We validated the pathway analysis findings by demonstrating the positive correlation between PGAM1 expression and the expression of anti-apoptotic molecule Bcl-2, immune checkpoint molecule PD-L1(CD274), and EMT-inducing molecule Snail1 in the TCGA dataset (**Figure 6D**). As shown in **Figure 7A**, the protein expression of PGAM1 is located in the cytoplasm. The molecular docking analysis of these proteins with PGAM1 (PDB: 4gwg) showed that PD-L1 (PDB: 5dxw) interacted with PGAM1 (PDB: 4gwg), and the interacting amino acids included GLN-46, LYS-45, GLU-91, ARG-47, GLU-48, ASP-64, ASP-104, LEU-49, ARG-67, PRO-101, TRY-39, ASN-61, ASP-58. Snail (PDB: 5xbu) interconnects with PGAM1 (PDB: 4gwg), and the amino acid sites are ASP-58, LYS-80, ASP-79, HIS-51, GLU-78, ASN-82, ILE-81, LYS-124, PHE-69, TRP-128, ARG-126, ASP-119, SER-123, ILE-130, SER-127, GLU-115, GLU-9, TYR-12, ASP-8, MET-6. BCl2 (PDB: 5tzp) interconnects with PGAM1 (PDB: 4gwg), and the amino acid sites are GLU-159, PRO-161, ARG-17, GLU-158, ARG-78, PRO-18, PHE-14, ASN-15, PRO-6, GLN-5, LEU-3, PRO-87, PHE-85 (**Figure 7B**). Taken together, our results unveil PGAM1 as a candidate regulator of both apoptosis and immune response in UVM. We speculate that the interplay between these two PGAM1-related cascades may cooperatively drive UVM pathogenesis. Further investigations are necessitated to dissect the intricate mechanisms linking PGAM1 signaling to apoptosis and immune pathways in the context of UVM advancement.

### PGAM1 was highly expressed in UVM tissues and cell lines and was positively correlated with poor prognosis

**Figure 8:**
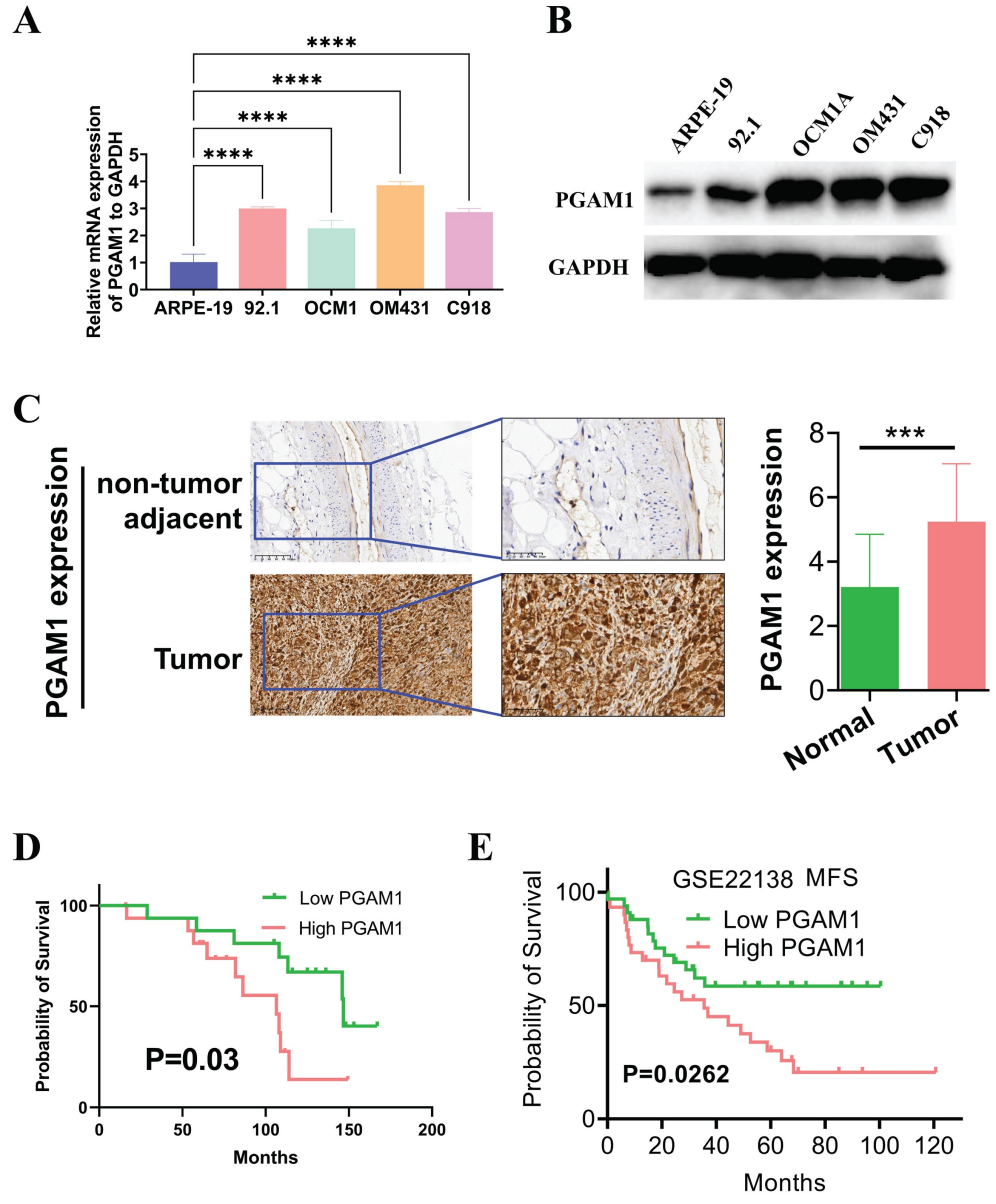
Detection of PGAM1 expression level in UVM cells and tissues. (A) Western blotting and (B) qRT-PCR were used to detect the expression level of PGAM1 in uveal melanoma (UVM) cell lines (92.1, OCM1A, OM431 and C918) and human retinal pigment epithelial (RPE) cell line (ARPE-19).** (C)** Immunohistochemistry was used to analyze the protein levels of PGAM1 in 33 pairs of uveal melanoma and adjacent normal control tissue. **(D)** Kaplan-Meier was used to analyze the correlation between PGAM1 and OS of UVM patients. (E) Kaplan-Meier analysis of PGAM1 expression and MFS in GSE22138 UVM cohort.

In this study, the expression pattern of PGAM1 was systematically examined in cell lines using western blotting, and quantitative real-time PCR. Four UVM cell lines were compared with normal human retinal pigment epithelial cells, and higher expression of PGAM1 was found at both mRNA and protein levels in the UVM lines (**Figures 8A and 8B**). To validate the clinical relevance of these findings, we collected UVM 33 pairs tumor samples and adjacent normal tissue for immunohistochemical analysis. Consistent with the cell line data, elevated PGAM1 protein expression was detected in UVM tumors compared to normal control tissues (**Figure 8C**). Further, Kaplan-Meier analysis showed a significant negative correlation between high expression of PGAM1 and OS of in our collection of UVM patients (**Figure 8D**). In a separate GEO UVM cohort, it was also confirmed that UVM patients with higher PGAM1 expression had a worse prognosis, with MFS (metastasis-free survival) significantly lower in the high expression group than in the low expression group (**Figure 8E**). This further corroborates that PGAM1 overexpression is associated with UVM pathogenesis. In conclusion, our study demonstrates that PGAM1 promotes UVM pathogenesis by increasing the migratory and invasive capacities of UVM cells, while inhibiting apoptosis and immune surveillance. Therefore, PGAM1 may be a potential therapeutic target for UVM treatment.

### PGAM1 promotes UVM pathogenesis by enhancing migration, invasion and immune evasion, while inhibiting apoptosis

**Figure 9:**
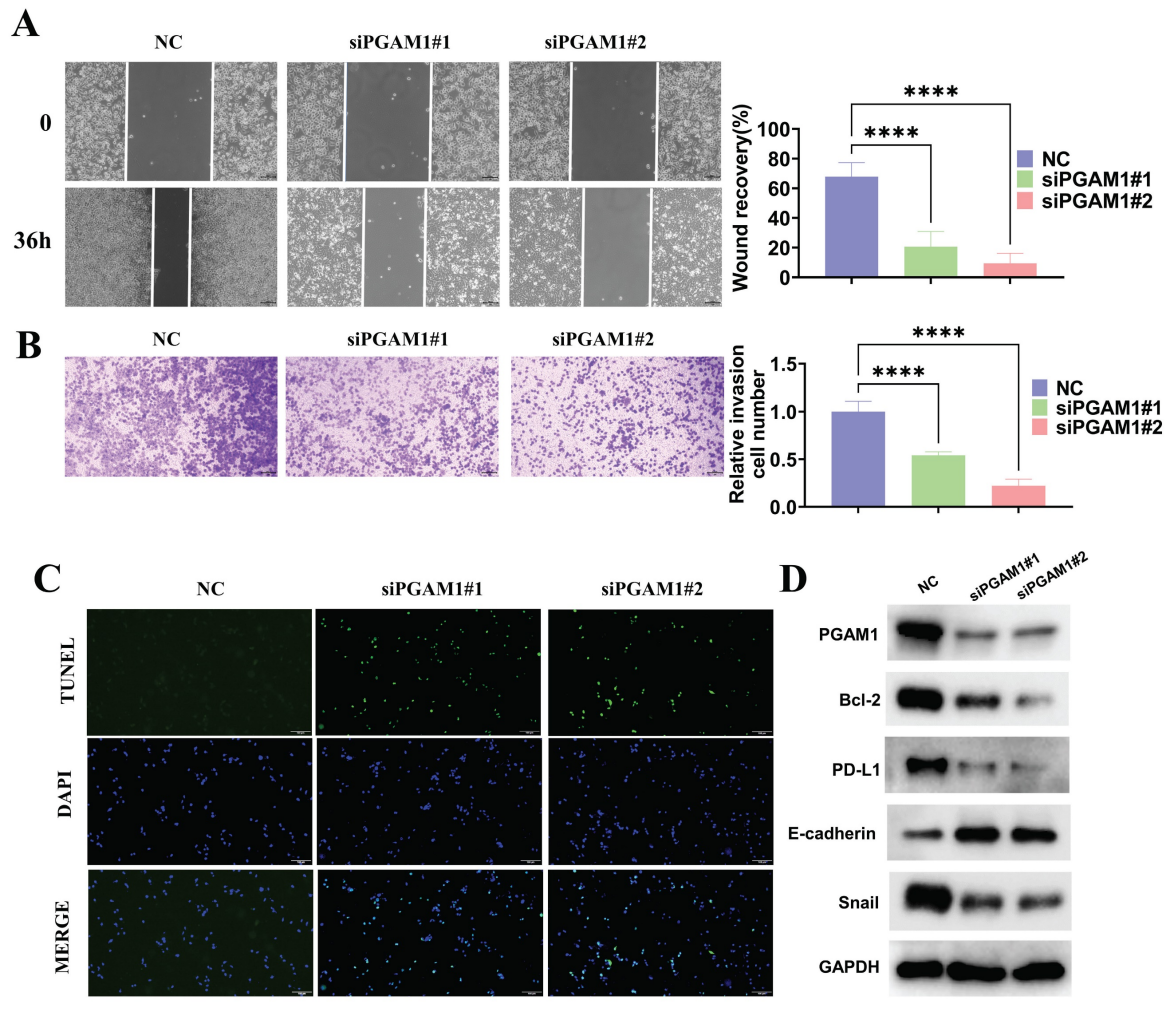
Knockdown of PGAM1 expression inhibits cells migration and invasion and promote cell apoptosis. Scratch wound healing assay (A) and transwell invasion assay (B) were used to examine the migration and invasion abilities of PGAM1-silenced C918 cells, respectively. (C) TUNEL assay was utilized to evaluate the apoptotic level of C918 cells following PGAM1 knockdown. (D) Western blot analysis of PGAM1, Bcl-2, PD-L1, Snail and E-cadherin expression following PGAM1 knockdown.

To investigate the function of PGAM1 in the uveal melanoma cell line C918, the expression of PGAM1 was inhibited using small interfering RNA (siRNA) and CCK8, scratch wound healing, transwell invasion and TUNEL assays were employed to detect its effects on cells. The results showed that inhibition of PGAM1 led to significantly reduced the proliferative, migratory and invasive abilities of C918 cells and an increased occurrence of apoptosis (**Supplementary Figure 9** and **Figure 9A-9C**). Moreover, we detected changes in the expression of several molecules related to epithelial-mesenchymal transition (EMT), apoptosis and immune evasion in PGAM1 knockdown cells. Specifically, In PGAM1 knockdown cells, the immune checkpoint molecule PD-L1. mesenchymal marker Snail and the anti-apoptosis Bcl-2 were significantly decreased, whereas the epithelial marker E-cadherin were increased (**Figures 9D**).

## Discussion

PGAM1 is a key enzyme involved in glycolysis, and its expression level and functional regulation in a variety of tumors have received extensive attention 41, 42. In this study, we discussed the expression and function of PGAM1 in tumors and its possible regulatory mechanisms from multiple perspectives. We found that the expression of PGAM1 in a variety of solid tumors was significantly higher than that in normal tissues and was closely associated with tumor progression and clinical prognosis. We also found that PGAM1 expression is affected by epigenetic mechanisms and may be an important regulator of tumor metabolism. First, we systematically analyzed the expression patterns of PGAM1 in different tumor types using PGAM1 mRNA expression profile microarray data from 33 solid tumors in the TCGA-GTEx and TCGA databases. We found that PGAM1 expression was significantly higher in the majority of solid tumor tissues than in normal tissues. This result is highly consistent with the report of Hitosugi et al 41, indicating that the expression of PGAM1 in tumors is universal and specific. To verify whether the expression of PGAM1 mRNA was consistent with the changes in protein levels, we further analyzed the expression and prognosis of PGAM1 protein in tumors using the HPA database and found that PGAM1 was highly expressed in 9 types of tumors and associated with poor prognosis.

Meanwhile, we also used the UALCAN online database 43 to analyze the methylation level of PGAM1 and the post-translational modification level of PGAM1 protein, and confirmed that the high expression of PGAM1 mRNA and protein was consistent with the decrease of DNA methylation level and the increase of protein phosphorylation level. The expression pattern was consistent with the changes in protein levels detected by mass spectrometry in the group of Mellet et al 44. These data suggest that PGAM1 expression in tumors is regulated at multiple levels, possibly reflecting the high demand for PGAM1 by tumor cells.

We analyzed the relationship between PGAM1 expression level and patients' clinical prognosis, survival curve and K-M Cox regression model, and found that high expression of PGAM1 was significantly correlated with poor survival, which was an independent prognostic factor, suggesting that PGAM1 could be a potential diagnostic target. This is highly similar to the negative correlation between PGAM1 and OS in NSCLC, OSCC, glioma and BRCA. In glioma, PGAM1 expression is associated with increased mortality and shorter OS 45. In NSCLC, PGAM1 expression is associated with reduced overall and progression-free survival, as well as distant and lymph node metastasis 46. In oral squamous cell carcinoma (OSCC), PGAM1 expression is associated with tumor recurrence, lymphatic metastasis and poor OS 47. In breast cancer, PGAM1 expression is also reported to be a negative prognostic factor 14.

These studies suggest that the expression level of PGAM1 can be used as a prognostic indicator for tumor patients, and also provides a basis for targeted tumor therapy. We also found that patients with high PGAM1 mRNA expression were often accompanied by elevated tumor grading status, suggesting that PGAM1 may be involved in regulating tumor progression. This hypothesis is in line with existing studies that have reported that inhibition of PGAM1 significantly suppresses proliferation and induces apoptosis in glioblastoma 48. These studies suggest that PGAM1 may be an important metastasis-promoting gene that is closely related to tumor progression and clinical prognosis.

PGAM1 is an important rate-limiting enzyme of the glycolytic pathway, and its involvement in the regulation of energy metabolism in tumor cells has been widely reported 41. However, the potential upstream regulatory mechanism of PGAM1 expression itself has rarely been reported. In this study, we systematically evaluated the methylation level of PGAM1 in the UALCAN database and found that the methylation level of PGAM1 was significantly reduced in eight cancer types. This finding suggests that PGAM1 may act as a downstream target gene that is activated and regulated at the epigenetic level. DNA methylation is known to play a key role in development and disease by influencing the methylation pattern of CpG islands and even genome-wide, which can be involved in the precise regulation of gene expression 49. A large body of evidence suggests that hypomethylation of tumor oncogene regions and hypermethylation of oncogenes jointly promote tumor development 50. The association of PGAM1 hypomethylation with the development of various solid tumors, including BLCA, CHOL, LIHC, LUAD, PRAD, READ, UCEC and TGCT, was first discovered by our laboratory. More importantly, our correlation analysis highlighted a high positive correlation between a number of methylation-related epigenetic regulators, including DNA methyltransferases such as DNMT1, DNMT3A and DNMT3B, as well as other methylation modifications such as m1A, m5C and m6A, and the expression level of PGAM1. Therefore, we hypothesized that there is a cascade network connecting the activation of upstream methylation enzymes and downstream effector molecules of PGAM1 51, 52. However, whether there are key methylation sites in this regulatory network that directly regulate PGAM1 expression, whether the detection of PGAM1 hypomethylation can be used as a tumor marker, and the effect of altering its methylation status on the behavior of tumors need to be further elucidated in the future by molecular editing and phenotyping methods. In conclusion, our study suggests that epigenetic regulation may somehow be involved in mediating the aberrant activation of PGAM1 in a variety of tumors.

Tumor microenvironment (TME) as the overall environment that supports tumor growth and development, the composition and functional status of different immune cell subsets play a key role in tumor occurrence, progression, metastasis and the restriction of therapeutic response 53. In this paper, we systematically analyzed the relationship between PGAM1 expression and tumor infiltration of various immune cells. The results showed that PGAM1 expression was positively correlated with CD4+ T cells, CD8+ T cells cells. This suggests that PGAM1 may be widely involved in regulating the differentiation and activation of immune cells in TME. Tumor -associated macrophages (TAMs) and other M2-type macrophages can promote tumor angiogenesis, enhance cancer stemness, inhibit CTL cytotoxic T lymphocyte activity by secreting IL-10, TGF-β and other cytokines and enzymes, thus achieving immune suppression and creating an immune escape microenvironment for tumor cells 54. And we found that PGAM1 positively regulated TAMs tumor infiltration, suggesting that there may be positive feedback between them.

PD-L1 plays a crucial immunoregulatory role by suppressing T cell activation, thus preventing autoimmunity. Downregulation of PD-L1 may therefore be a mechanism to enhance anti-tumor immunity 55, 56. In addition, we also found a clear positive correlation between PGAM1 expression and immune checkpoint molecule PD-L1. PD-L1, as an inhibitory receptor on the surface of tumor cells, binds to PD-1 on the surface of T lymphocytes, activates downstream signaling pathways to inhibit T lymphocyte proliferation and cytotoxicity 57, and has been considered as one of the “don't eat me” signals. We subsequently found that PGAM1 promoted PD-L1 protein expression in UVM. Therefore, PGAM1 may be directly involved in inhibiting tumor immune clearance by up-regulating PD-L1. Moreover, we found that PGAM1 expression was correlated with TMB and MSI in more than 10 types of tumors, and these two factors have been reported to be important biomarkers for predicting ICI efficacy and prognosis 58, 59, which further supports the potential link between PGAM1 and tumor immunity. Therefore, this study constructed a frame of multiple immune mechanisms in TME regulated by PGAM1 as the core. In summary, PGAM1 may affect tumor infiltration of TAMs and NK cells and other cells, up-regulate PD-L1 and ultimately achieve inhibition of CTLs and NK cells immune cytotoxic clearance function. And this mechanism of changing TME immune status creates an immune escape environment for tumor cells. In addition, PGAM1 involvement in regulating MSI/TMB status can also affect ICI efficacy. Therefore, PGAM1 may be a key immune modulator in TME, widely involved in regulating tumor immune status. This lays a theoretical foundation for developing PGAM1-related immune therapy strategies.

In this study, we tried to investigate the relationship between PGAM1 and tumor immune status and analyzed the expression correlation between PGAM1 and 23 immune checkpoint molecules by using the TCGA database. The results showed a positive or inverse correlation between PGAM1 expression and ICMs such as CD274/PD-L1 in a variety of solid tumor samples including UVM. We further confirmed that PGAM1 was mainly localized in the cytoplasm and might promote the expression of PD-L1 protein in UVM using immunohistochemistry and WB methods. Correlation analysis showed that PGAM1 was widely and positively correlated with a variety of cancer-promoting immune cells such as TAMs, NK cells, and mDCs. Moreover, patients with high PGAM1 expression were positively correlated with immunotherapy response. Together, these results support the hypothesis that PGAM1 may intervene in the tumor immune microenvironment and tumor formation by regulating the functional status of multiple immune cells. Indeed, changes in different immune cell communities in the TME have been shown to influence tumor progression and metastasis and therapeutic response 60. In the future, we can continue to analyze the downstream immune profiles regulated by PGAM1 through single cell sequencing and other technologies. In addition, we reasonably hypothesize that PGAM1 promotion of immune checkpoint molecules, such as PD-L1, in UVMs may facilitate their escape from host immunosurveillance, but the specific mechanisms and intermediate processes here remain to be explored in situ. In addition, we analyzed the intrinsic connection between PGAM1 and MSI/TMB, two predictive markers for tumor immunotherapy. Although the possible role of PGAM1 in immunotherapeutic resistance and the brand-new CAR-T technology system has yet to be supported by experimental data, as a widespread pro-tumor effector molecule, it is significant to study its intrinsic relevance in tumor immune status and clinical translational effects.

CeRNA regulatory network, as a gene expression regulation mechanism based on the miRNA-dependent binding and interaction among competitive endogenous RNA molecules, has been found to be widely involved in various physiological and pathological processes. In our study, the upstream miRNA regulators of PGAM1 were predicted by bioinformatics methods, and a PGAM1-related ceRNA regulatory network containing multiple non-coding RNAs was established. CeRNA regulatory network is a mechanism that regulates gene expression by the competitive binding and interaction among endogenous RNA molecules dependent on miRNA, and it plays an important role in various physiological and pathological processes 61. Our analysis found that the 3'-UTR region of PGAM1 could complementarily pair with multiple tumor-related miRNAs, and these miRNAs could competitively bind with multiple lncRNA and circRNA molecules 33. Therefore, we speculated that the endogenous competition in this ceRNA network might have a regulatory effect on the expression level of PGAM1 as a downstream target gene. In fact, more and more evidence has shown that ceRNA network plays a key role in the occurrence and development of various tumors, and the abnormalities in the network might lead to the overexpression of oncogenes or the low expression of tumor suppressor genes 62. Our study constructed a PGAM1-centered ceRNA regulatory network and provided a new perspective for deeply understanding its expression regulation mechanism.

Further in-depth analysis revealed enrichment of PGAM1-correlated genes and pathways in critical oncogenic processes encompassing GLYCOLYSIS, EMT, apoptosis and INFLAMMATORY RESPONSE. As a key glycolytic enzyme, PGAM1 may rewire cancer metabolism by enhancing aerobic glycolysis to fulfill the anabolic and energetic demands of uncontrolled cancer growth and metastasis 8, 9, 63, 64. Moreover, PGAM1 may also contribute to EMT, invasion and metastasis by modulating the Wnt/β-catenin signaling pathway in pancreatic ductal adenocarcinoma (PDAC), as well as by affecting the cytoskeletal dynamics through the small Rho GTPase pathways 65, 66. In addition, PGAM1 silencing inhibited HCC cell proliferation in vitro and tumor growth in vivo, and enhanced CD8+ T cell infiltration in an ferroptosis manne 67. Consistent with these results, we demonstrated that PGAM1 silencing suppressed the migratory and invasive capacities of UVM cells. Cancer-related biological processes such as EMT, apoptosis, and immune response were significantly activated in PGAM1-high UVM patient samples, as shown by GSEA enrichment analysis. The positive correlation between PGAM1 expression and its downstream targets CD274, Snail1, and Bcl2 expression was validated by GEO dataset, and the inhibition of these targets' expression by PGAM1 knockdown was confirmed by WB assay. The anti-apoptotic protein Bcl-2 inhibits programmed cell death through interference with multiple apoptotic pathways, enabling prolonged cell survival 67. The expression of E-cadherin, a crucial marker of epithelial cells, is reduced in epithelial-mesenchymal transition (EMT), a phenomenon that confers cancer cells with greater motility and invasiveness. Transcription factors such as Snail mediate EMT by binding to the E-box sequences in the E-cadherin promoter and inhibiting its transcription 64, 65. Our results demonstrated that PGAM1 knockdown led to upregulation of the EMT marker E-cadherin and downregulation of the anti-apoptotic marker Bcl-2, the immune checkpoint molecule PD-L1, and the mesenchymal marker Snail. These results suggest that the invasion, metastasis, and anti-apoptosis ability of UVM cells may be enhanced by PGAM1 through regulating the activity of these targets. Moreover, the distribution of PGAM1 in the cytoplasm and its direct binding to PD-L1, Snail, Bcl2, and other key signal molecules in UVM were revealed by immunofluorescence and molecular docking techniques. This finding aligns with previous reports where PGAM1 was shown to have multifaceted roles beyond its metabolic activity, influencing various cancer-related biological processes. For instance, PGAM1 has been implicated in the activation of DNA damage repair pathways, as shown in gliomas where it interacts with Wip1, inhibiting its translocation into the nucleus and consequently attenuating the ATM signaling pathway. This non-enzymatic activity of PGAM1 highlights its potential as a theranostic target in cancer treatment strategies. Additionally, apart from its role in metabolism, PGAM1 interacts with ACTA2 in breast cancer cells, driving motility and metastasis independent of its metabolic activity by promoting actin filament assembly 68, 69. This underscores PGAM1's role in cell motility and metastasis, providing a broader understanding of its impact in cancer biology.

Ubiquitination, a process that tags proteins for degradation, is a central mechanism in cellular regulation. Given the crucial role of post-translational modifications (PTMs) in protein function and stability, we propose that PGAM1 may influence the ubiquitination levels of PD-L1, Bcl-2, and Snail. This modification could result in altered protein stability, leading to changes in their abundance. The ubiquitination process, which tags proteins for degradation, is a pivotal mechanism in cellular regulation, and its modulation by PGAM1 could significantly impact the roles of these proteins in apoptosis (Bcl-2), immune evasion (PD-L1), and EMT (Snail). The direct binding of PGAM1 to these proteins, as indicated by our molecular docking results, suggests that PGAM1 may be involved in stabilizing or destabilizing these protein complexes. Such interactions could influence the signaling pathways and biological processes in which these proteins are involved, thereby affecting their functional efficacy in UVM cells. PGAM1's interaction with these key molecules might also affect various downstream signaling pathways. For example, by modulating Bcl-2, PGAM1 could influence apoptotic pathways, while its interaction with Snail could impact the EMT process, a critical step in cancer metastasis. In summary, a PGAM1-centered UVM signal network was established by our study, and valuable clues for understanding its complex relationship with EMT, apoptosis, and immune microenvironment were provided.

The important role of PGAM1 in UVM progression was revealed by our study, and its potential as a clinical biomarker or therapeutic target was evaluated. PGAM1 was found to be significantly overexpressed in four UVM cell lines by us, implying that its overexpression might be a key factor for UVM cells to acquire malignant phenotype. To verify this hypothesis, a PGAM1 knockdown UVM cell model was constructed by us, and the inhibition of UVM cell migration, and invasion, the promotion of cell apoptosis, and the alteration of EMT and immune checkpoint processes by PGAM1 silencing were demonstrated. This indicated the function of PGAM1 as a UVM driver factor. Therefore, PGAM1, as a cytoplasmic signal molecule with clear localization and active function, might be an excellent therapeutic target for UVM. In fact, effective targeted drugs were lacked by UVM currently, resulting in poor prognosis for late or metastatic UVM. Our study provided a new idea for UVM precision medicine. In addition, the overexpression of PGAM1 protein in UVM patient tumor tissues was also confirmed by us by immunohistochemistry, suggesting that PGAM1 might be used for the clinical detection and prognosis evaluation of UVM. However, the in-depth mechanism of PGAM1 from molecular phenotype to tumor formation, and the safety and efficacy evaluation of PGAM1-targeted therapy strategy still need to be further verified by experimental techniques.

## Conclusions

In summary, our comprehensive study demonstrated that PGAM1 overexpression is associated with unfavorable outcomes and promotes UVM progression through immune evasion, EMT, and apoptosis. This suggests that PGAM1 is a valuable prognostic biomarker and a potential therapeutic target for personalized treatment of UVM patients.

## Supplementary Material

Supplementary figures and tables.

## Figures and Tables

**Table 1:** PGAM1 expression distribution for Pre-treatment and On-treatment samples in all datasets

No.	Study	Cancers	Anti Target	Response Mean	Non-Response Mean	Log2FC	FDR	Value of P
**1**	SRP183455; SRP217040	NSCLC	anti-PD1/PDL1	4,565.00	3,204.00	0.671	0.11	0.011
**2**	ERP107734	Gastric Cancer	anti-PD1	4,467.50	3,234.00	0.35	0.145	0.02
**3**	SRP217040	Non-small Cell Lung Cancer	anti-PDL1	4,661.00	3,612.00	0.824	0.188	0.018
**4**	SRP150548	Melanoma	anti-PD1	292	2,414.50	-1.927	0.362	0.034
**5**	ERP105482; SRP150548; SRP128156	Melanoma, RCC	anti-PD1 + anti-CTLA4	4,237.00	3,396.00	0.377	0.42	0.065
**6**	SRP011540	Melanoma	anti-PD1	2,310.00	2,551.50	0.166	0.449	0.157
**7**	anti-PD1	Melanoma, NSCLC, GBM, RCC, GC	anti-PD1	3,132.50	3,194.50	0.077	0.497	0.251
**8**	ERP105482	Melanoma	anti-PD1 + anti-CTLA4	4,113.00	4,071.00	0.338	0.612	0.152
**9**	ERP105482; SRP011540; SRP070710; SRP094781; SRP150548; SRP230414; SRP250849; SRP302761	Melanoma	anti-PD1/anti-CTLA4/anti-PD1 + anti-CTLA4	3,042.00	2,843.50	0.057	0.656	0.409
**10**	SRP183455	Non-small Cell Lung Cancer	anti-PD1	2,706.00	2,790.00	0.372	0.713	0.385
**11**	SRP011540	Melanoma	anti-CTLA4	3,835.00	3,096.00	0.393	0.76	0.12
**12**	SRP011540; SRP150548; SRP302761	Melanoma	anti-CTLA4	3,835.00	2,805.00	0.393	0.778	0.118
**13**	SRP230414	Melanoma	anti-PD1	6,482.50	6,992.00	-0.28	0.791	0.29
**14**	SRP070710	Melanoma	anti-PD1	7,335.00	7,157.50	-0.233	0.83	0.41
**15**	GSE67501	Renal Cell Carcinoma	anti-PD1	11.11	11.48	-0.369	0.879	0.156
**16**	IMvigor210	Urothelial Cancer	anti-PDL1	18.01	17.5	-0.046	0.884	0.763
**17**	SRP128156	RCC	anti-PD1/anti-PD1 + anti-CTLA4	6,053.50	6,937.00	-0.483	0.888	0.178
**18**	GSE111636	Urothelial Cancer	anti-PD1	4.4	4.33	0.073	0.922	0.543
**19**	SRP155030	Glioblastoma	anti-PD1	3,462.50	3,396.50	0.106	0.926	0.611
**20**	PMID: 33806963I	Renal Cell Carcinoma	anti-PD1	13.23	12.78	0.451	0.942	0.266
**21**	GSE176307	Urothelial Cancer	anti-PD1	10.3	10.37	-0.066	0.961	0.728
**22**	PMID: 32472114	Renal Cell Carcinoma	anti-PD1	34.52	34.45	0.071	0.981	0.627
**23**	GSE122220	Melanoma	anti-PD1	6.2	6.6	-0.401	0.992	0.637
**24**	TCGA	Melanoma	anti-CTLA4	11.72	11.96	-0.24	0.996	0.51
**25**	SRP250849	Melanoma	anti-PD1	1,560.83	2,609.67	-0.121	0.998	0.784
**26**	SRP094781	Melanoma	anti-PD1	2,491.50	2,674.00	-0.001	0.999	0.997
**27**	GSE99070	Malignant Pleural Mesothelioma	anti-PD1	6.76	6.77	-0.014	1	0.848
**28**	SRP128156	Renal Cell Carcinoma	anti-PD1 + anti-CTLA4	5,715.00	2,873.00	0.42	1	0.553
**29**	SRP128156	Renal Cell Carcinoma	anti-PD1	6,662.00	6,983.50	-0.473	1	0.323
**30**	ERP105482	Melanoma	anti-PD1	2,509.00	3,082.50	0	1	0.999

## References

[1] Siegel RL, Miller KD, Wagle NS (2023). Cancer statistics, 2023. Ca Cancer J Clin.

[2] Bray F, Ferlay J, Soerjomataram I (2018). Global cancer statistics 2018: GLOBOCAN estimates of incidence and mortality worldwide for 36 cancers in 185 countries. Ca Cancer J Clin.

[3] Kaliki S, Shields CL (2017). Uveal melanoma: relatively rare but deadly cancer. Eye (Lond).

[4] McLaughlin CC, Wu XC, Jemal A (2005). Incidence of noncutaneous melanomas in the U.S. Cancer.

[5] Chen X, Wu Q, Depeille P (2017). RasGRP3 Mediates MAPK Pathway Activation in GNAQ Mutant Uveal Melanoma. Cancer Cell.

[6] Jarczak J, Karska-Basta I, Romanowska-Dixon B (2023). Deterioration of Visual Acuity after Brachytherapy and Proton Therapy of Uveal Melanoma, and Methods of Counteracting This Complication Based on Recent Publications. Medicina.

[7] Kujala E, Mäkitie T, Kivelä T (2003). Very long-term prognosis of patients with malignant uveal melanoma. Invest Ophthalmol Vis Sci.

[8] Pavlova NN, Thompson CB (2016). The Emerging Hallmarks of Cancer Metabolism. Cell Metab.

[9] Liberti MV, Locasale JW (2016). The Warburg Effect: How Does it Benefit Cancer Cells?. Trends Biochem Sci.

[10] Hitosugi T, Zhou L, Elf S (2012). Phosphoglycerate mutase 1 coordinates glycolysis and biosynthesis to promote tumor growth. Cancer Cell.

[11] Yang GJ, Tao F, Zhong HJ (2022). Targeting PGAM1 in cancer: An emerging therapeutic opportunity. Eur J Med Chem.

[12] Jiang X, Sun Q, Li H (2014). The role of phosphoglycerate mutase 1 in tumor aerobic glycolysis and its potential therapeutic implications. Int J Cancer.

[13] Li D, Cheng X, Zheng W (2020). Glucosamine-6-Phosphate Isomerase 1 Promotes Tumor Progression and Indicates Poor Prognosis in Hepatocellular Carcinoma. Cancer Manag Res.

[14] Liu M, Li R, Wang M (2022). PGAM1 regulation of ASS1 contributes to the progression of breast cancer through the cAMP/AMPK/CEBPB pathway. Mol Oncol.

[15] Li F, Yang H, Kong T (2020). PGAM1, regulated by miR-3614-5p, functions as an oncogene by activating transforming growth factor-β (TGF-β) signaling in the progression of non-small cell lung carcinoma. Cell Death Dis.

[16] Wen CL, Huang K, Jiang LL (2019). An allosteric PGAM1 inhibitor effectively suppresses pancreatic ductal adenocarcinoma. Proc Natl Acad Sci U S a.

[17] Ren F, Wu H, Lei Y (2010). Quantitative proteomics identification of phosphoglycerate mutase 1 as a novel therapeutic target in hepatocellular carcinoma. Mol Cancer.

[18] Qu J, Sun W, Zhong J (2017). Phosphoglycerate mutase 1 regulates dNTP pool and promotes homologous recombination repair in cancer cells. J Cell Biol.

[19] Zhang D, Jin N, Sun W (2017). Phosphoglycerate mutase 1 promotes cancer cell migration independent of its metabolic activity. Oncogene.

[20] Luo J, Yang T, Wu J (2023). Exosomal PGAM1 promotes prostate cancer angiogenesis and metastasis by interacting with ACTG1. Cell Death Dis.

[21] Feng Y, Zhang X, Zhang S (2022). PGAM1 Promotes Glycolytic Metabolism and Paclitaxel Resistance via Pyruvic Acid Production in Ovarian Cancer Cells. Front Biosci.

[22] Laurent C, Valet F, Planque N (2011). High PTP4A3 phosphatase expression correlates with metastatic risk in uveal melanoma patients. Cancer Res.

[23] Uhlén M, Fagerberg L, Hallström BM (2015). Proteomics. Tissue-based map of the human proteome. Science.

[24] Liu J, Lichtenberg T, Hoadley KA (2018). An integrated TCGA pan-cancer clinical data resource to drive high-quality survival outcome analytics. Cell.

[25] Shen W, Song Z, Zhong X (2022). Sangerbox: A comprehensive, interaction-friendly clinical bioinformatics analysis platform. iMeta.

[26] Thorsson V, Gibbs DL, Brown SD (2018). The Immune Landscape of Cancer. Immunity.

[27] Bonneville R, Krook MA, Kautto EA (2017). Landscape of microsatellite instability across 39 cancer types. Jco Precis Oncol.

[28] Badal VD, Kundrotas PJ, Vakser IA (2021). Text mining for modeling of protein complexes enhanced by machine learning. Bioinformatics.

[29] Singh A, Copeland MM, Kundrotas PJ (2023). GRAMM Web Server for Protein Docking. Computational Drug Discovery and Designed.

[30] Singh A, Dauzhenka T, Kundrotas PJ (2020). Application of docking methodologies to modeled proteins. Proteins: Structure, Function, and Bioinformatics.

[31] Liu W, Wang X (2019). Prediction of functional microRNA targets by integrative modeling of microRNA binding and target expression data. Genome Biol.

[32] Chen Y, Wang X (2020). miRDB: an online database for prediction of functional microRNA targets. Nucleic Acids Res.

[33] Li JH, Liu S, Zhou H (2014). starBase v2.0: decoding miRNA-ceRNA, miRNA-ncRNA and protein-RNA interaction networks from large-scale CLIP-Seq data. Nucleic Acids Res.

[34] Xie Q, Ding J, Chen Y (2021). Role of CD8+ T lymphocyte cells: Interplay with stromal cells in tumor microenvironment. Acta Pharmaceutica Sinica B.

[35] Liu T, Han C, Wang S (2019). Cancer-associated fibroblasts: an emerging target of anti-cancer immunotherapy. Journal of hematology & oncology.

[36] Choucair K, Morand S, Stanbery L (2020). TMB: a promising immune-response biomarker, and potential spearhead in advancing targeted therapy trials. Cancer Gene Ther.

[37] van Velzen M, Derks S, van Grieken N (2020). MSI as a predictive factor for treatment outcome of gastroesophageal adenocarcinoma. Cancer Treat Rev.

[38] Yang M, Miao YR, Xie GY (2022). ICBatlas: A Comprehensive Resource for Depicting Immune Checkpoint Blockade Therapy Characteristics from Transcriptome Profiles. Cancer Immunol Res.

[39] Salmena L, Poliseno L, Tay Y (2011). A ceRNA hypothesis: the Rosetta Stone of a hidden RNA language?. Cell.

[40] Palucka A K, Coussens L M (2016). The basis of oncoimmunology. Cell.

[41] Weng Y, Duan W, Yu X, Wu F, Yang D, Jiang Y, Wu J, Wang M, Wang X, Shen Y, Zhang Y, Xu H (2023). MicroRNA-324-3p inhibits osteosarcoma progression by suppressing PGAM1-mediated aerobic glycolysis. Cancer Sci.

[42] Vander Heiden MG, Locasale JW, Swanson KD (2010). Evidence for an alternative glycolytic pathway in rapidly proliferating cells. Science.

[43] Chandrashekar DS, Bashel B, Balasubramanya SAH (2017). UALCAN: a portal for facilitating tumor subgroup gene expression and survival analyses. Neoplasia.

[44] Gong T, Jiang Y, Shao C (2022). Proteome-centric cross-omics characterization and integrated network analyses of triple-negative breast cancer. Cell Rep.

[45] Gao H, Yu B, Yan Y (2013). Correlation of expression levels of ANXA2, PGAM1, and CALR with glioma grade and prognosis. J Neurosurg.

[46] Sun Q, Li S, Wang Y (2018). Phosphoglyceric acid mutase-1 contributes to oncogenic mTOR-mediated tumor growth and confers non-small cell lung cancer patients with poor prognosis. Cell Death & Differentiation.

[47] Zhang D, Wu H, Zhang X (2017). Phosphoglycerate mutase 1 predicts the poor prognosis of oral squamous cell carcinoma and is associated with cell migration. J Cancer.

[48] Zhang A, Tao W, Zhai K (2020). Protein sumoylation with SUMO1 promoted by Pin1 in glioma stem cells augments glioblastoma malignancy. Neuro Oncol.

[49] Dawson VL, Dawson TM, Kang S (2023). DNA Methylation signature of aging: Potential impact on the pathogenesis of parkinson's disease. Journal of Parkinson's Disease.

[50] Davalos V, Esteller M (2023). Cancer epigenetics in clinical practice. CA: a cancer journal for clinicians.

[51] Man X, Li Q, Wang B (2022). DNMT3A and DNMT3B in breast tumorigenesis and potential therapy. Front Cell Dev Biol.

[52] Wong KK (2021). DNMT1: A key drug target in triple-negative breast cancer. Semin Cancer Biol.

[53] Hänggi K, Ruffell B (2023). Cell death, therapeutics, and the immune response in cancer. Trends Cancer.

[54] Li Q, Mei A, Qian H (2023). The role of myeloid-derived immunosuppressive cells in cardiovascular disease. Int Immunopharmacol.

[55] Ingram JR, Dougan M, Rashidian M (2017). PD-L1 is an activation-independent marker of brown adipocytes. Nat Commun.

[56] Yamaguchi H, Hsu J, Yang W (2022). Mechanisms regulating PD-L1 expression in cancers and associated opportunities for novel small-molecule therapeutics. Nat Rev Clin Oncol.

[57] Wang D, Wu X, Sun Y (2022). Therapeutic targets and biomarkers of tumor immunotherapy: Response versus non-response. Signal Transduct Target Ther.

[58] Guo W, Jin L, Liang J (2022). Detection of mutation profiles and tumor mutation burden of cerebrospinal fluid circulating DNA by a cancer genomic panel sequencing in glioma patients. Clin Chim Acta.

[59] Kwon M, An M, Klempner SJ (2021). Determinants of Response and Intrinsic Resistance to PD-1 Blockade in Microsatellite Instability-High Gastric Cancer. Cancer Discov.

[60] Obeid E, Nanda R, Fu Y (2013). The role of tumor-associated macrophages in breast cancer progression. Int J Oncol.

[61] Tay Y, Rinn J, Pandolfi PP (2014). The multilayered complexity of ceRNA crosstalk and competition. Nature.

[62] Karreth FA, Tay Y, Perna D (2011). In vivo identification of tumor-suppressive PTEN ceRNAs in an oncogenic BRAF-induced mouse model of melanoma. Cell.

[63] Kim D, Khin PP, Lim OK (2022). LPA/LPAR1 signaling induces PGAM1 expression via AKT/mTOR/HIF-1α pathway and increases aerobic glycolysis, contributing to keratinocyte proliferation. Life Sci.

[64] An X, Li T, Chen N (2021). PGAM1 regulates the glycolytic metabolism of SCs in tibetan sheep and its influence on the development of SCs. Gene.

[65] Ghanavat M, Shahrouzian M, Deris ZZ (2021). Digging deeper through glucose metabolism and its regulators in cancer and metastasis. Life Sci.

[66] Liu X, Tan X, Liu P (2018). Phosphoglycerate Mutase 1 (PGAM1) Promotes Pancreatic Ductal Adenocarcinoma (PDAC) Metastasis by Acting as a Novel Downstream Target of the PI3K/Akt/mTOR Pathway. Oncol Res.

[67] Zheng Y, Wang Y, Lu Z (2023). PGAM1 Inhibition Promotes HCC Ferroptosis and Synergizes with Anti-PD-1 Immunotherapy. Adv Sci (Weinh).

[68] Johannessen TA, Mukherjee J (2021). Phosphoglycerate mutase 1 (PGAM1) overexpression promotes radio- and chemoresistance in gliomas by activating the DNA damage response. Mol Cell Oncol.

[69] Ohba S, Johannessen TA, Chatla K (2020). Phosphoglycerate mutase 1 activates DNA damage repair via regulation of WIP1 activity. Cell Rep.

