# Supplementary material for: Pan-Cancer Analysis of PGAM1 and Its Experimental Validation in Uveal Melanoma Progression
Source: J Cancer. 2024 Feb 17;15(7):2074–94. doi: 10.7150/jca.93398 (PMC10905406; doi:10.7150/jca.93398)
Supplement: Supplementary file 1 — Supplementary figures and tables. [file jcav15p2074s1.pdf]

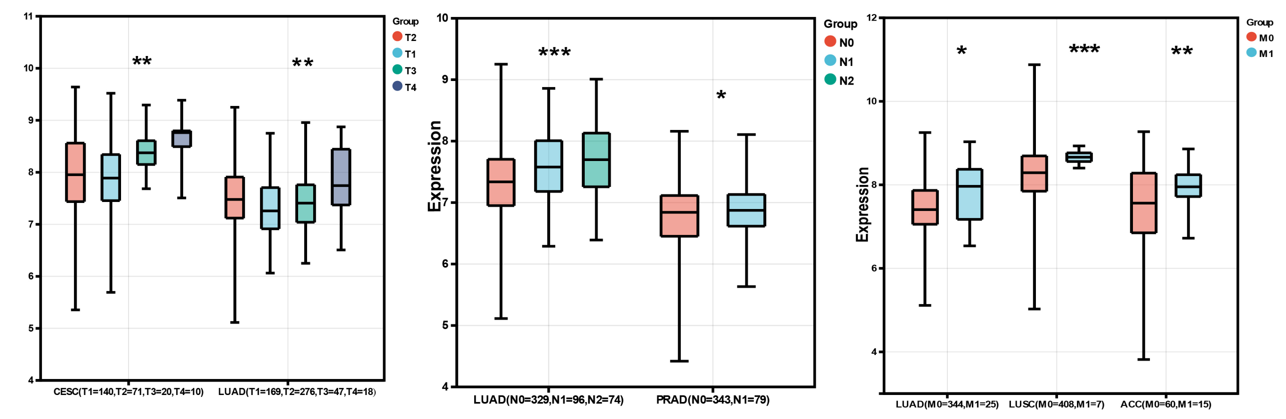

Group
T2
T1
T3
T4
**
**
CESC(T1=140,T2=71,T3=20,T4=10)
LUAD(T1=169,T2=276,T3=47,T4=18)
Expression
Group
N0
N1
N2
***
*
LUAD(N0=329,N1=96,N2=74)
PRAD(N0=343,N1=79)
Expression
Group
M0
M1
*
***
**
LUAD(M0=344,M1=25)
LUSC(M0=408,M1=7)
ACC(M0=60,M1=15)

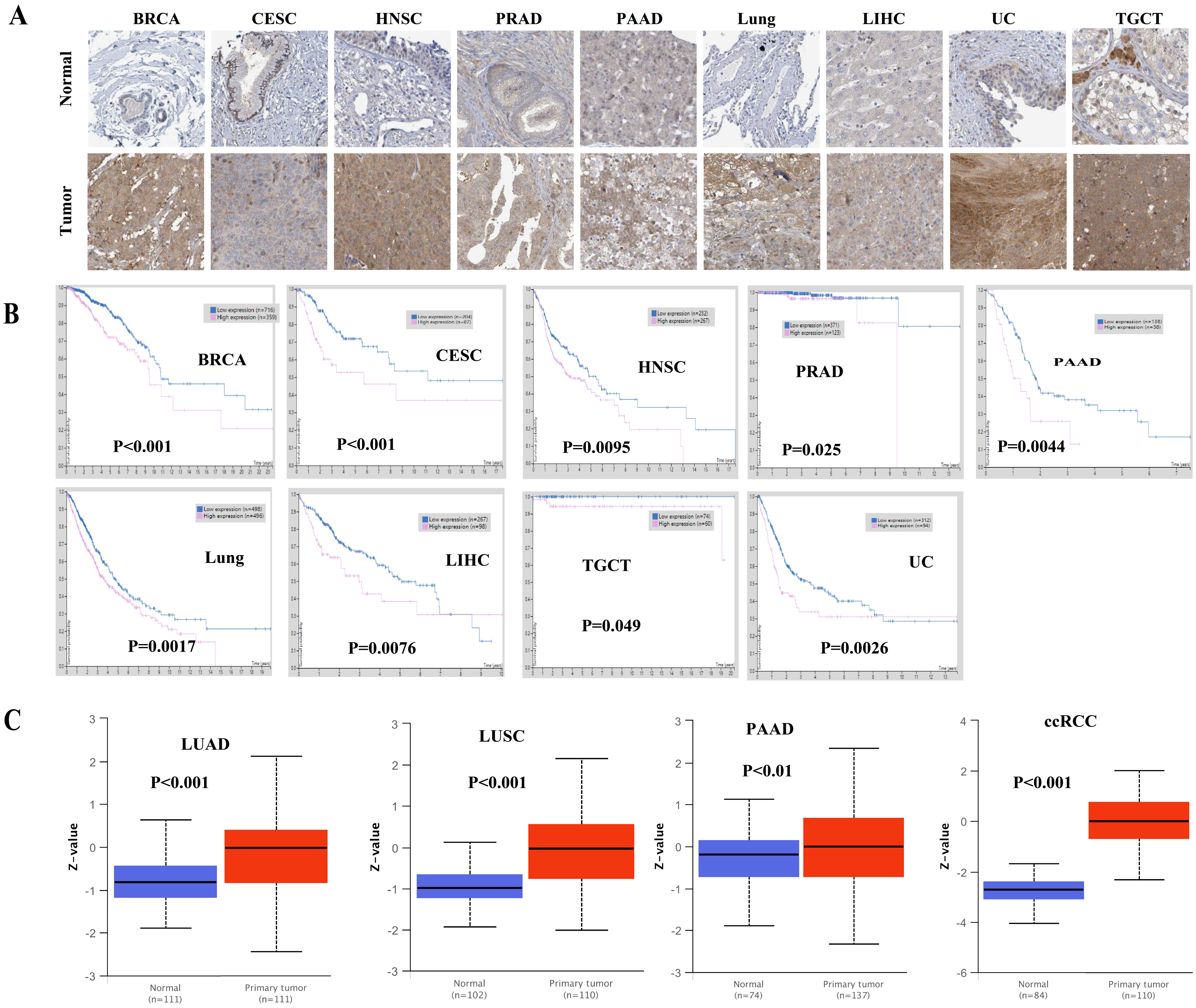

A
BRCA
CESC
HNSC
PRAD
PAAD
Lung
LIHC
UC
TGCT
Normal
Tumor
B
Low expression (n=716)
High expression (n=359)
BRCA
P<0.001
CESC
P<0.001
Low expression (n=232)
High expression (n=267)
HNSC
P=0.0095
Low expression (n=371)
High expression (n=123)
PRAD
P=0.025
Low expression (n=138)
High expression (n=38)
PAAD
P=0.0044
Low expression (n=498)
High expression (n=496)
Lung
P=0.0017
Low expression (n=267)
High expression (n=98)
LIHC
P=0.0076
Low expression (n=74)
High expression (n=60)
TGCT
P=0.049
Low expression (n=312)
High expression (n=94)
UC
P=0.0026
Survival probability
Time (years)
C
LUAD
P<0.001
Z-value
Normal
(n=111)
Primary tumor
(n=111)
LUSC
P<0.001
Normal
(n=102)
Primary tumor
(n=110)
PAAD
P<0.01
Normal
(n=74)
Primary tumor
(n=137)
ccRCC
P<0.001
Normal
(n=84)
Primary tumor
(n=110)

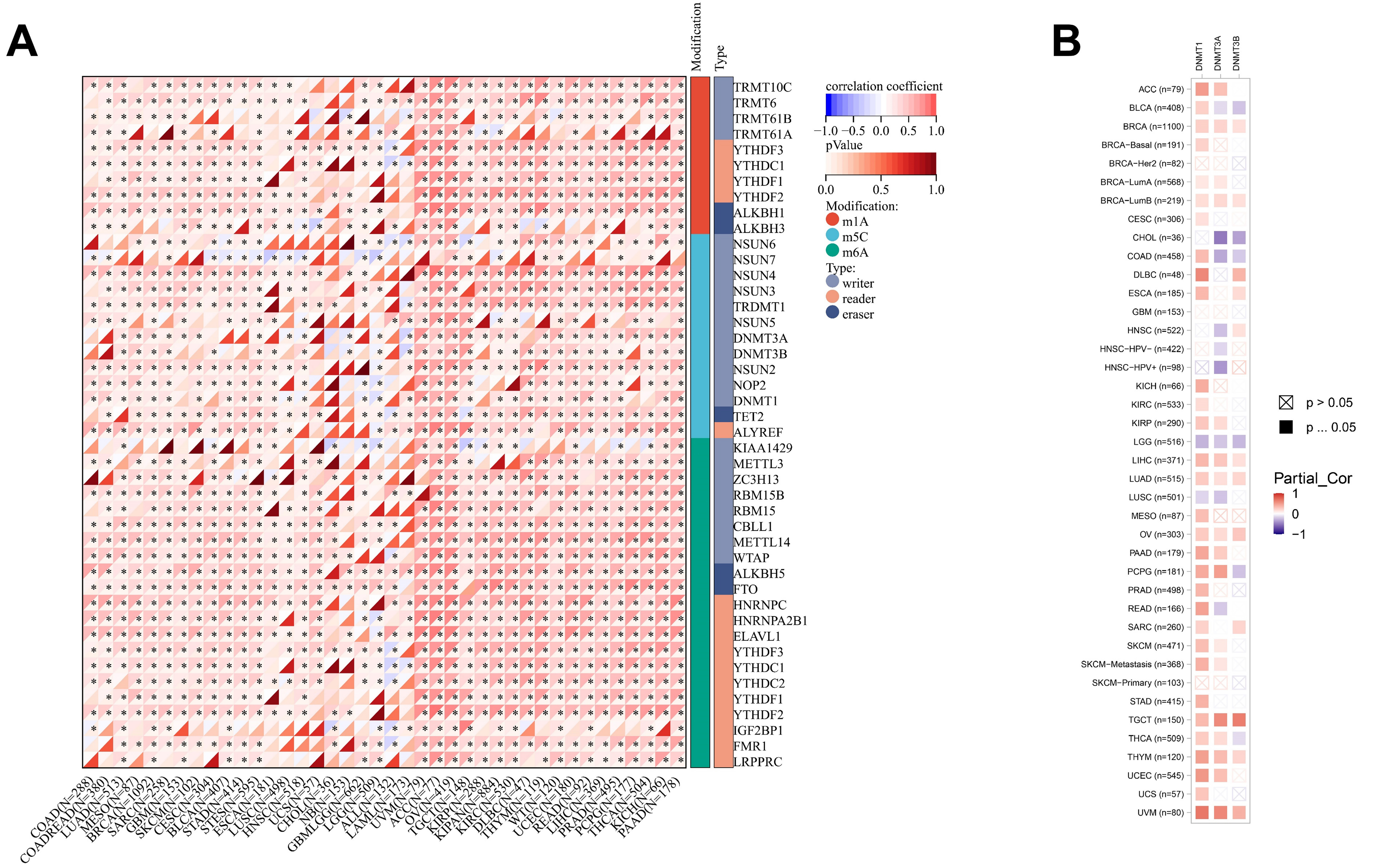
A
Modification
Type
TRMT10C
TRMT6
TRMT61B
TRMT61A
YTHDF3
YTHDC1
YTHDF1
YTHDF2
ALKBH1
ALKBH3
NSUN6
NSUN7
NSUN4
NSUN3
TRDMT1
NSUN5
DNMT3A
DNMT3B
NSUN2
NOP2
DNMT1
TET2
ALYREF
KIAA1429
METTL3
ZC3H13
RBM15B
RBM15
CBLL1
METTL14
WTAP
ALKBH5
FTO
HNRNPC
HNRNPA2B1
ELAVL1
YTHDF3
YTHDC1
YTHDC2
YTHDF1
YTHDF2
IGF2BP1
FMR1
LRPPRC
correlation coefficient
−1.0 −0.5 0.0 0.5 1.0
pValue
0.0 0.5 1.0
Modification:
m1A
m5C
m6A
Type:
writer
reader
eraser
COAD(N=288)
COADREAD(N=380)
LUAD(N=513)
MESO(N=87)
BRCA(N=1092)
SARC(N=258)
GBM(N=153)
SKCM(N=102)
CESC(N=304)
BLCA(N=407)
STAD(N=414)
STES(N=595)
ESCA(N=181)
LUSC(N=498)
HNSC(N=518)
UCS(N=57)
CHOL(N=36)
NB(N=153)
GBMLGG(N=662)
LGG(N=509)
ALL(N=132)
LAML(N=173)
UVM(N=79)
ACC(N=77)
OV(N=419)
TGCT(N=148)
KIRP(N=288)
KIPAN(N=884)
KIRC(N=530)
DLBC(N=47)
THYM(N=119)
WT(N=120)
UCEC(N=180)
READ(N=92)
LIHC(N=369)
PRAD(N=495)
PCPG(N=177)
THCA(N=504)
KICH(N=66)
PAAD(N=178)
B
DNMT1
DNMT3A
DNMT3B
ACC (n=79)
BLCA (n=408)
BRCA (n=1100)
BRCA−Basal (n=191)
BRCA−Her2 (n=82)
BRCA−LumA (n=568)
BRCA−LumB (n=219)
CESC (n=306)
CHOL (n=36)
COAD (n=458)
DLBC (n=48)
ESCA (n=185)
GBM (n=153)
HNSC (n=522)
HNSC−HPV− (n=422)
HNSC−HPV+ (n=98)
KICH (n=66)
KIRC (n=533)
KIRP (n=290)
LGG (n=516)
LIHC (n=371)
LUAD (n=515)
LUSC (n=501)
MESO (n=87)
OV (n=303)
PAAD (n=179)
PCPG (n=181)
PRAD (n=498)
READ (n=166)
SARC (n=260)
SKCM (n=471)
SKCM−Metastasis (n=368)
SKCM−Primary (n=103)
STAD (n=415)
TGCT (n=150)
THCA (n=509)
THYM (n=120)
UCEC (n=545)
UCS (n=57)
UVM (n=80)
p > 0.05
p ... 0.05
Partial_Cor
1
0
−1

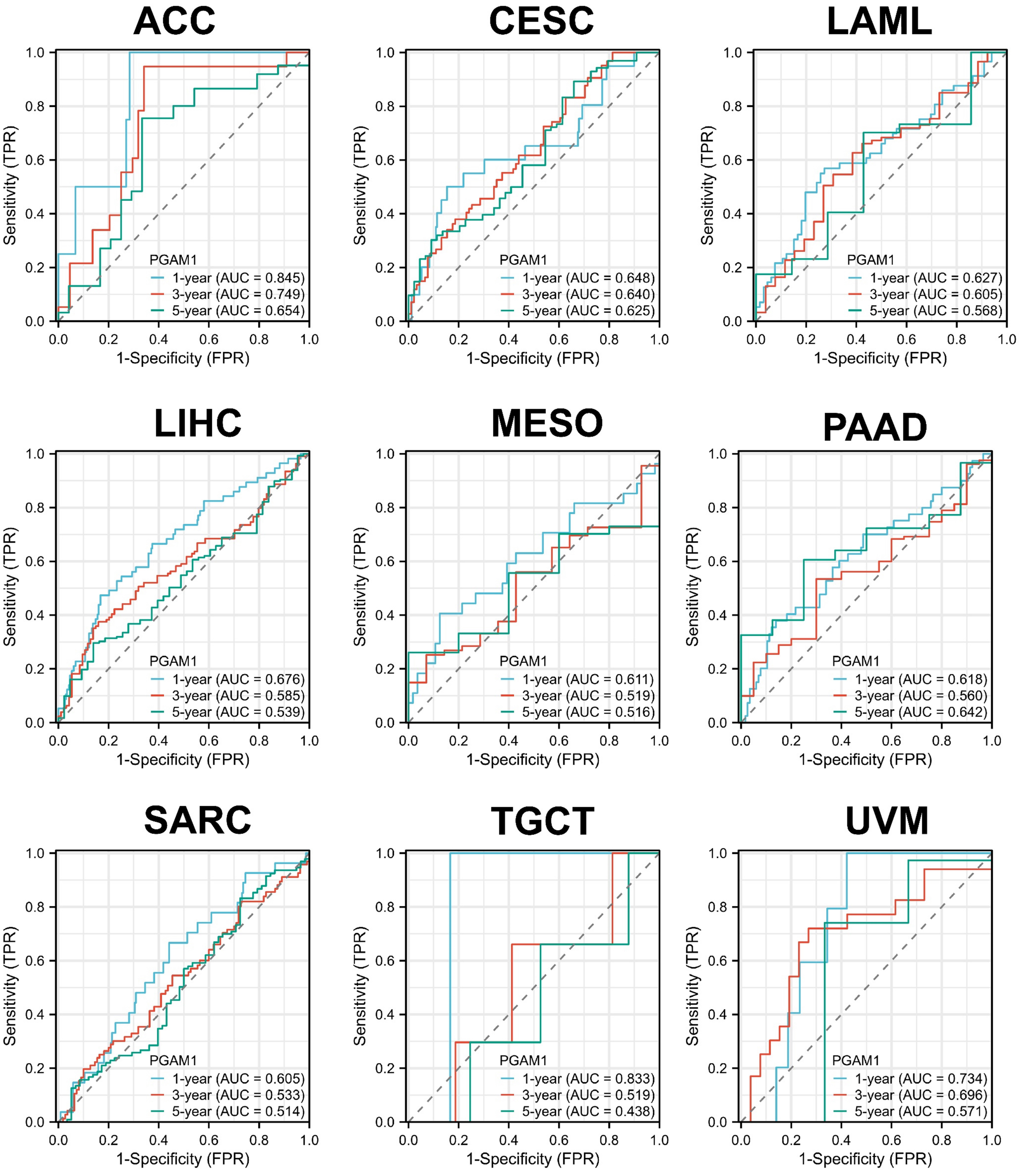

ACC
Sensitivity (TPR)
1-Specificity (FPR)
PGAM1
1-year (AUC = 0.845)
3-year (AUC = 0.749)
5-year (AUC = 0.654)
CESC
Sensitivity (TPR)
1-Specificity (FPR)
PGAM1
1-year (AUC = 0.648)
3-year (AUC = 0.640)
5-year (AUC = 0.625)
LAML
Sensitivity (TPR)
1-Specificity (FPR)
PGAM1
1-year (AUC = 0.627)
3-year (AUC = 0.605)
5-year (AUC = 0.568)
LIHC
Sensitivity (TPR)
1-Specificity (FPR)
PGAM1
1-year (AUC = 0.676)
3-year (AUC = 0.585)
5-year (AUC = 0.539)
MESO
Sensitivity (TPR)
1-Specificity (FPR)
PGAM1
1-year (AUC = 0.611)
3-year (AUC = 0.519)
5-year (AUC = 0.516)
PAAD
Sensitivity (TPR)
1-Specificity (FPR)
PGAM1
1-year (AUC = 0.618)
3-year (AUC = 0.560)
5-year (AUC = 0.642)
SARC
Sensitivity (TPR)
1-Specificity (FPR)
PGAM1
1-year (AUC = 0.605)
3-year (AUC = 0.533)
5-year (AUC = 0.514)
TGCT
Sensitivity (TPR)
1-Specificity (FPR)
PGAM1
1-year (AUC = 0.833)
3-year (AUC = 0.519)
5-year (AUC = 0.438)
UVM
Sensitivity (TPR)
1-Specificity (FPR)
PGAM1
1-year (AUC = 0.734)
3-year (AUC = 0.696)
5-year (AUC = 0.571)

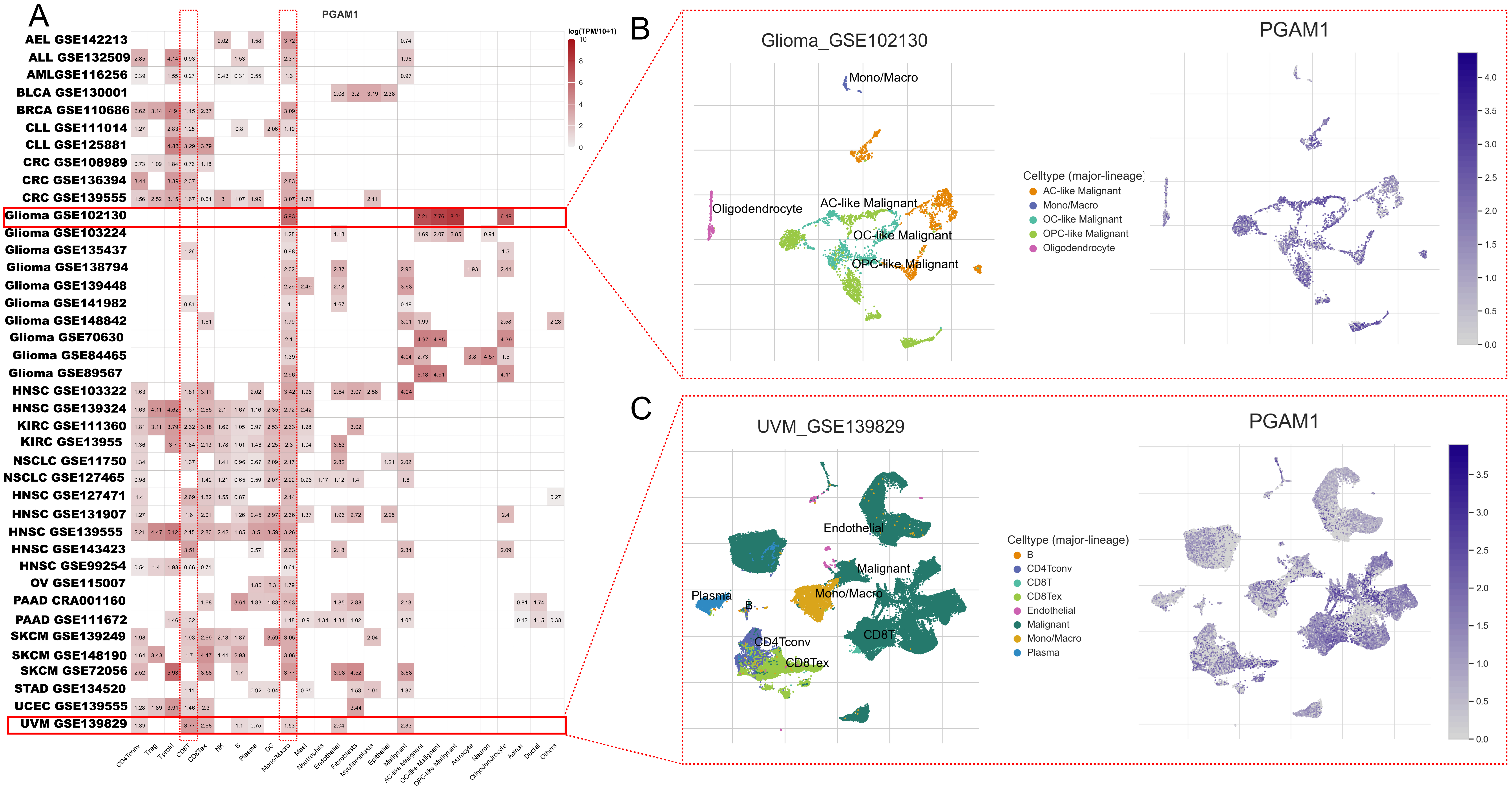
A
PGAM1
log(TPM/10+1)
AEL GSE142213
ALL GSE132509
AMLGSE116256
BLCA GSE130001
BRCA GSE110686
CLL GSE111014
CLL GSE125881
CRC GSE108989
CRC GSE136394
CRC GSE139555
Glioma GSE102130
Glioma GSE103224
Glioma GSE135437
Glioma GSE138794
Glioma GSE139448
Glioma GSE141982
Glioma GSE148842
Glioma GSE70630
Glioma GSE84465
Glioma GSE89567
HNSC GSE103322
HNSC GSE139324
KIRC GSE111360
KIRC GSE13955
NSCLC GSE11750
NSCLC GSE127465
HNSC GSE127471
HNSC GSE131907
HNSC GSE139555
HNSC GSE143423
HNSC GSE99254
OV GSE115007
PAAD CRA001160
PAAD GSE111672
SKCM GSE139249
SKCM GSE148190
SKCM GSE72056
STAD GSE134520
UCEC GSE139555
UVM GSE139829
CD4Tconv
Treg
Tprolif
CD8T
CD8Tex
NK
B
Plasma
DC
Mono/Macro
Mast
Neutrophils
Endothelial
Fibroblasts
Myofibroblasts
Epithelial
Malignant
AC-like Malignant
OC-like Malignant
OPC-like Malignant
Astrocyte
Neuron
Oligodendrocyte
Acinar
Ductal
Others
B
Glioma_GSE102130
Mono/Macro
Oligodendrocyte
AC-like Malignant
OC-like Malignant
OPC-like Malignant
Celltype (major-lineage)
AC-like Malignant
Mono/Macro
OC-like Malignant
OPC-like Malignant
Oligodendrocyte
PGAM1
C
UVM_GSE139829
Endothelial
Malignant
Plasma
B
Mono/Macro
CD8T
CD4Tconv
CD8Tex
Celltype (major-lineage)
B
CD4Tconv
CD8T
CD8Tex
Endothelial
Malignant
Mono/Macro
Plasma
PGAM1

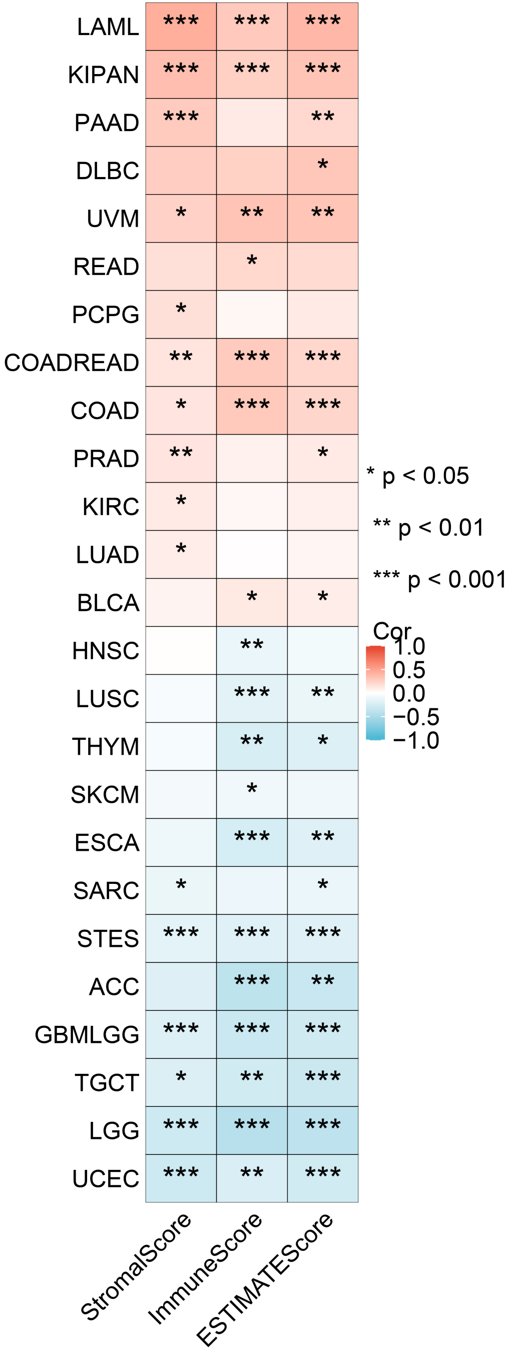
LAML
KIPAN
PAAD
DLBC
UVM
READ
PCPG
COADREAD
COAD
PRAD
KIRC
LUAD
BLCA
HNSC
LUSC
THYM
SKCM
ESCA
SARC
STES
ACC
GBMLGG
TGCT
LGG
UCEC
StromalScore
ImmuneScore
ESTIMATEScore
* p < 0.05
** p < 0.01
*** p < 0.001
Cor
1.0
0.5
0.0
−0.5
−1.0

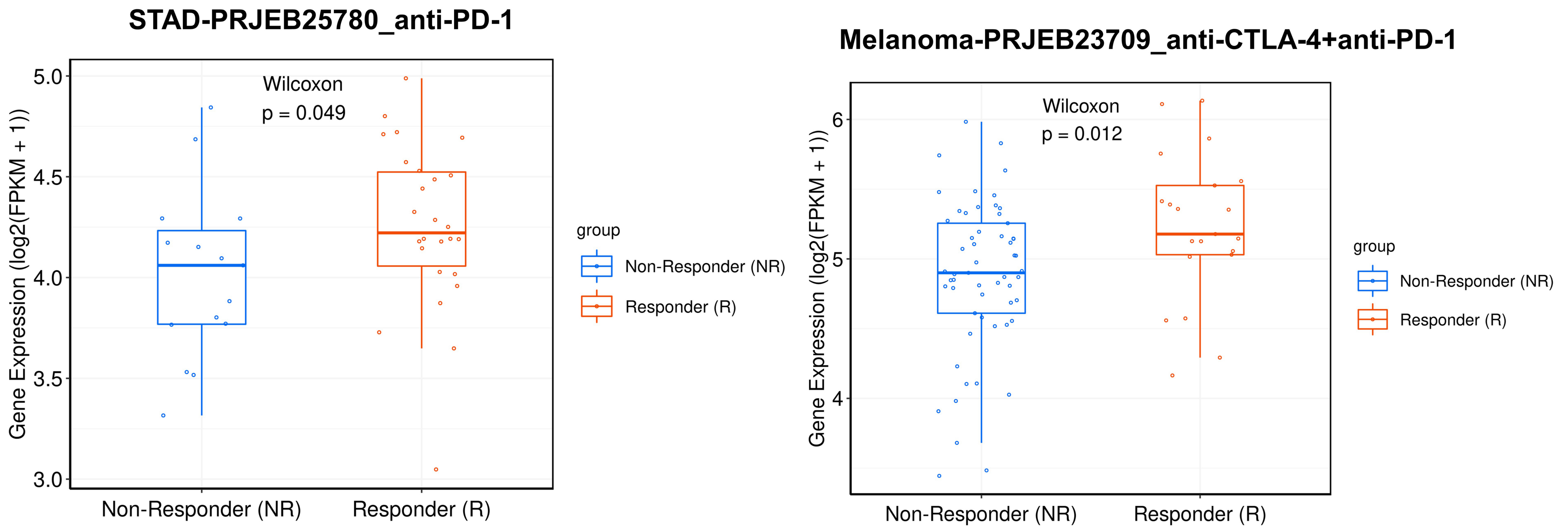

STAD-PRJEB25780_anti-PD-1
Wilcoxon
p = 0.049
Gene Expression (log2(FPKM + 1))
5.0
4.5
4.0
3.5
3.0
Non-Responder (NR)
Responder (R)
group
Non-Responder (NR)
Responder (R)
Melanoma-PRJEB23709_anti-CTLA-4+anti-PD-1
Wilcoxon
p = 0.012
Gene Expression (log2(FPKM + 1))
6
5
4
Non-Responder (NR)
Responder (R)
group
Non-Responder (NR)
Responder (R)

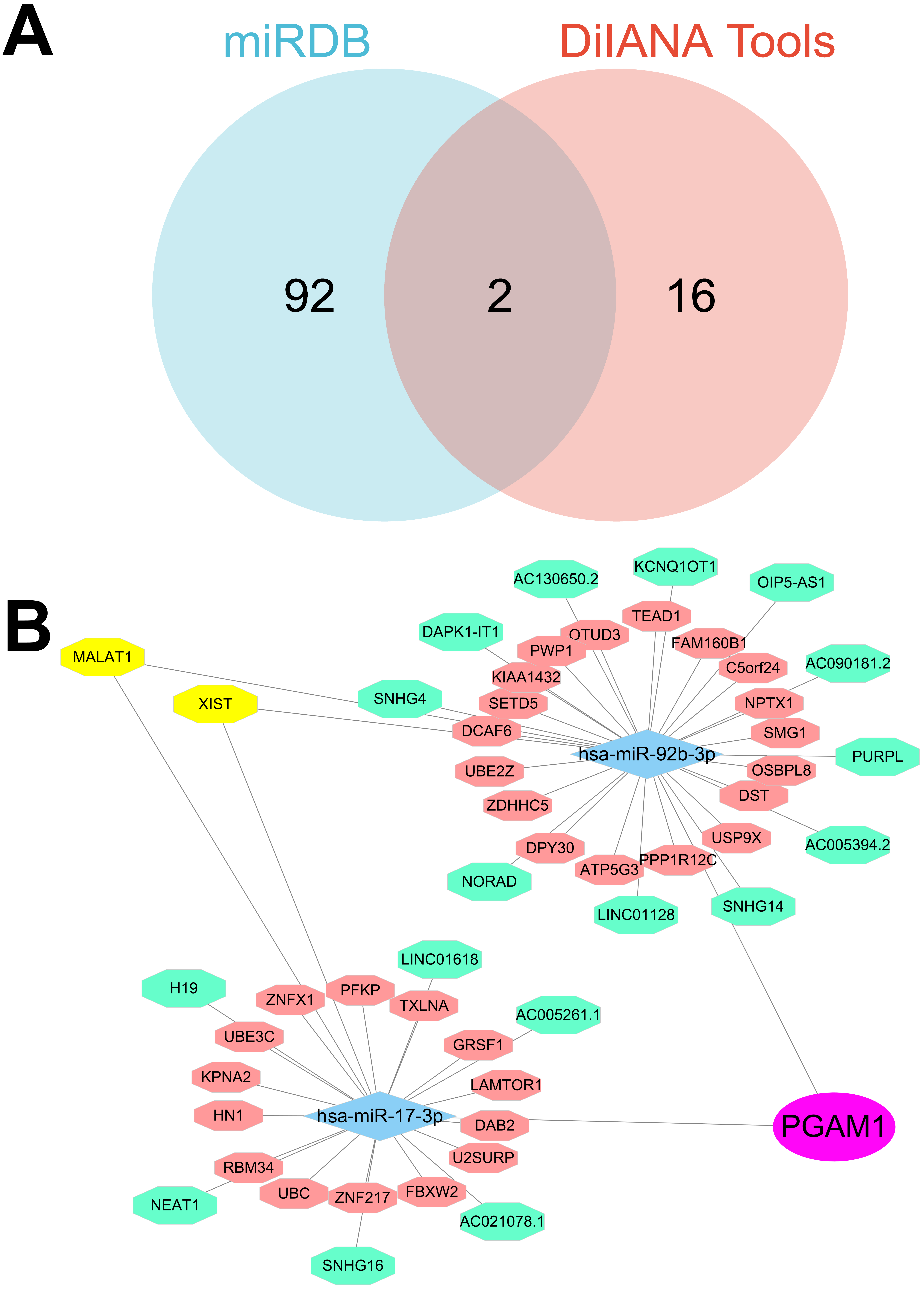
A
miRDB
DIIANA Tools
92
2
16
B
MALAT1
XIST
SNHG4
AC130650.2
KCNQ1OT1
OIP5-AS1
DAPK1-IT1
TEAD1
OTUD3
PWP1
FAM160B1
C5orf24
AC090181.2
KIAA1432
SETD5
NPTX1
DCAF6
SMG1
hsa-miR-92b-3p
PURPL
UBE2Z
OSBPL8
ZDHHC5
DST
USP9X
AC005394.2
DPY30
ATP5G3
PPP1R12C
NORAD
LINC01128
SNHG14
LINC01618
H19
ZNFX1
PFKP
TXLNA
AC005261.1
UBE3C
GRSF1
KPNA2
LAMTOR1
HN1
hsa-miR-17-3p
DAB2
PGAM1
U2SURP
RBM34
UBC
ZNF217
FBXW2
NEAT1
AC021078.1
SNHG16

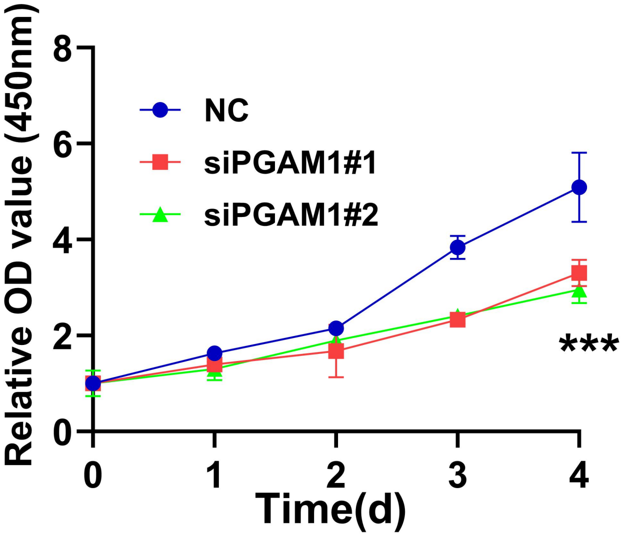

Relative OD value (450nm)
8
6
4
2
0
NC
siPGAM1#1
siPGAM1#2
***
0
1
2
3
4
Time(d)

Table1. Clinical significance analysis of PGAM1 in ACC

Characteristics	Low expression of PGAM1	High expression of PGAM1	P value
n	39	40	
Pathologic T stage, n (%)			0.117
T1	6 (7.8%)	3 (3.9%)	
T2	24 (31.2%)	18 (23.4%)	
T3	3 (3.9%)	5 (6.5%)	
T4	5 (6.5%)	13 (16.9%)	
Pathologic N stage, n (%)			0.168
N0	36 (46.8%)	32 (41.6%)	
N1	2 (2.6%)	7 (9.1%)	
Pathologic stage, n (%)			**0.047**
Stage I	6 (7.8%)	3 (3.9%)	
Stage II	22 (28.6%)	15 (19.5%)	
Stage III	7 (9.1%)	9 (11.7%)	
Stage IV	3 (3.9%)	12 (15.6%)	
Clinical M stage, n (%)			**0.011**
M0	35 (45.5%)	27 (35.1%)	
M1	3 (3.9%)	12 (15.6%)	
Primary therapy outcome, n			0.061
PD	5 (7.5%)	13 (19.4%)	
SD	1 (1.5%)	1 (1.5%)	
PR	1 (1.5%)	0 (0%)	
CR	29 (43.3%)	17 (25.4%)	
Age, n (%)			0.732
<= 50	21 (26.6%)	20 (25.3%)	
> 50	18 (22.8%)	20 (25.3%)	

Table2. Clinical significance analysis of PGAM1 in CEAC

Characteristics	Low expression of PGAM1	High expression of PGAM1	P value
n	153	153	
Pathologic T stage, n (%)			**0.006**
T1	80 (32.9%)	60 (24.7%)	
T2	39 (16%)	33 (13.6%)	
T3&T4	8 (3.3%)	23 (9.5%)	
Pathologic N stage, n (%)			0.378
N0	75 (38.5%)	59 (30.3%)	
N1	30 (15.4%)	31 (15.9%)	
Pathologic M stage, n (%)			0.345
M0	56 (21.9%)	60 (23.4%)	
M1	4 (1.6%)	7 (2.7%)	
MX	71 (27.7%)	58 (22.7%)	
Clinical stage, n (%)			**0.005**
Stage I	94 (31.4%)	68 (22.7%)	
Stage II	34 (11.4%)	35 (11.7%)	
Stage III	15 (5%)	31 (10.4%)	
Stage IV	7 (2.3%)	15 (5%)	
Primary therapy outcome, n			0.105
PD	9 (4.1%)	14 (6.4%)	
SD	3 (1.4%)	3 (1.4%)	
PR	1 (0.5%)	7 (3.2%)	
CR	96 (43.8%)	86 (39.3%)	
Histological type, n (%)			**< 0.001**
Adenocarcinoma	42 (13.7%)	5 (1.6%)	
Adenosquamous	1 (0.3%)	5 (1.6%)	
Squamous cell carcinoma	110 (35.9%)	143 (46.7%)	
Histologic grade, n (%)			0.953
G1	9 (3.3%)	10 (3.6%)	
G2	70 (25.5%)	65 (23.7%)	
G3	63 (23%)	56 (20.4%)	
G4	1 (0.4%)	0 (0%)	

Characteristics	Low expression of PGAM1	High expression of PGAM1	P value
Age, n (%)			0.347
<= 50	98 (32%)	90 (29.4%)	
> 50	55 (18%)	63 (20.6%)	

Table3. Clinical significance analysis of PGAM1 in UVM

Characteristics	Low expression of PGAM1	High expression of PGAM1	P value
n	38	38	
Pathologic M stage, n			0.186
M0	27 (35.5%)	23 (30.3%)	
M1	0 (0%)	3 (3.9%)	
MX	11 (14.5%)	12 (15.8%)	
Pathologic stage, n			**0.018**
Stage II	24 (31.6%)	13 (17.1%)	
Stage III	14 (18.4%)	22 (28.9%)	
Stage IV	0 (0%)	3 (3.9%)	
Clinical T stage, n (%)			**0.018**
T2	2 (2.6%)	2 (2.6%)	
T3	23 (30.3%)	11 (14.5%)	
T4	13 (17.1%)	25 (32.9%)	
Clinical N stage, n (%)			1.000
N0	37 (48.7%)	36 (47.4%)	
NX	1 (1.3%)	2 (2.6%)	
Clinical M stage, n (%)			0.168
M0	37 (48.7%)	33 (43.4%)	
M1	0 (0%)	3 (3.9%)	
MX	1 (1.3%)	2 (2.6%)	
Clinical stage, n (%)			**0.005**
Stage II	24 (31.6%)	11 (14.5%)	
Stage III	14 (18.4%)	24 (31.6%)	
Stage IV	0 (0%)	3 (3.9%)	
Age, n (%)			0.491
<= 60	17 (22.4%)	20 (26.3%)	
> 60	21 (27.6%)	18 (23.7%)	

Table4. Clinical significance analysis of PGAM1 in LUAD

Characteristics	Low expression of PGAM1	High expression of PGAM1	P value
n	146	138	
Pathologic T stage, n (%)			**< 0.001**
T1	65 (22.9%)	27 (9.5%)	
T2	67 (23.6%)	90 (31.7%)	
T3&T4	14 (4.9%)	21 (7.4%)	
Pathologic N stage, n (%)			**0.003**
N0	110 (38.7%)	80 (28.2%)	
N1	25 (8.8%)	31 (10.9%)	
N2&N3	11 (3.9%)	27 (9.5%)	
Pathologic M stage, n (%)			**0.012**
M0	143 (50.4%)	126 (44.4%)	
M1	3 (1.1%)	12 (4.2%)	
Pathologic stage, n (%)			**< 0.001**
Stage I	95 (33.5%)	59 (20.8%)	
Stage II	33 (11.6%)	38 (13.4%)	
Stage III	15 (5.3%)	29 (10.2%)	
Stage IV	3 (1.1%)	12 (4.2%)	
Primary therapy outcome, n (%)			0.093
PD	21 (7.4%)	30 (10.6%)	
SD	17 (6%)	10 (3.5%)	
PR	3 (1.1%)	0 (0%)	
CR	105 (37%)	98 (34.5%)	
Age, n (%)			0.838
<= 65	79 (27.8%)	73 (25.7%)	
> 65	67 (23.6%)	65 (22.9%)	

Table5. Clinical significance analysis of PGAM1 in HNSC

Characteristics	Low expression of PGAM1	High expression of PGAM1	P value
n	252	252	
Pathologic T stage, n (%)			0.060
T1	27 (6%)	18 (4%)	
T2	73 (16.3%)	62 (13.8%)	
T3	39 (8.7%)	57 (12.7%)	
T4	77 (17.2%)	95 (21.2%)	
Pathologic N stage, n (%)			0.702
N0	78 (19%)	93 (22.6%)	
N1	34 (8.3%)	32 (7.8%)	
N2&N3	84 (20.4%)	90 (21.9%)	
Pathologic M stage, n (%)			1.000
M0	103 (54.5%)	85 (45%)	
M1	1 (0.5%)	0 (0%)	
Pathologic stage, n (%)			0.915
Stage I	11 (2.5%)	14 (3.2%)	
Stage II	33 (7.6%)	37 (8.5%)	
Stage III	40 (9.2%)	39 (8.9%)	
Stage IV	122 (28%)	140 (32.1%)	
Clinical T stage, n (%)			0.066
T1	18 (3.7%)	15 (3.1%)	
T2	84 (17.2%)	62 (12.7%)	
T3	55 (11.2%)	76 (15.5%)	
T4	86 (17.6%)	93 (19%)	
Clinical N stage, n (%)			0.671
N0	122 (25.3%)	118 (24.5%)	
N1	38 (7.9%)	42 (8.7%)	
N2	78 (16.2%)	77 (16%)	
N3	2 (0.4%)	5 (1%)	
Clinical M stage, n (%)			1.000
M0	234 (48.9%)	240 (50.1%)	
M1	2 (0.4%)	3 (0.6%)	
Clinical stage, n (%)			0.904

Characteristics	Low expression of PGAM1	High expression of PGAM1	P value
Stage I	9 (1.8%)	10 (2%)	
Stage II	50 (10.2%)	46 (9.4%)	
Stage III	48 (9.8%)	54 (11%)	
Stage IV	137 (28%)	136 (27.8%)	
Primary therapy outcome, n			0.160
PD	14 (3.3%)	27 (6.4%)	
SD	3 (0.7%)	3 (0.7%)	
PR	2 (0.5%)	4 (1%)	
CR	189 (45.1%)	177 (42.2%)	
Histologic grade, n (%)			0.356
G1	27 (5.6%)	35 (7.2%)	
G2	150 (31%)	151 (31.2%)	
G3	62 (12.8%)	57 (11.8%)	
G4	2 (0.4%)	0 (0%)	
Radiation therapy, n (%)			**0.035**
No	68 (15.4%)	87 (19.7%)	
Yes	156 (35.3%)	131 (29.6%)	

Table 6. Clinical significance analysis of PGAM1 in SKCM

Characteristics	Low expression of PGAM1	High expression of PGAM1	P value
n	155	166	
Pathologic T stage, n			0.668
T1	19 (5.9%)	18 (5.6%)	
T2	32 (10%)	37 (11.5%)	
T3	37 (11.5%)	48 (15%)	
T4	67 (20.9%)	63 (19.6%)	
Pathologic N stage, n			0.885
N0	91 (28.3%)	104 (32.4%)	
N1	29 (9%)	30 (9.3%)	
N2	16 (5%)	15 (4.7%)	
N3	19 (5.9%)	17 (5.3%)	
Pathologic M stage, n			0.086
M0	147 (45.8%)	164 (51.1%)	
M1	8 (2.5%)	2 (0.6%)	
Pathologic stage, n			0.158
Stage I	32 (10%)	39 (12.1%)	
Stage II	52 (16.2%)	64 (19.9%)	
Stage III	63 (19.6%)	61 (19%)	
Stage IV	8 (2.5%)	2 (0.6%)	
Radiation therapy, n			**0.014**
No	138 (43%)	131 (40.8%)	
Yes	17 (5.3%)	35 (10.9%)	
Age, n (%)			0.015
<= 60	92 (28.7%)	76 (23.7%)	
> 60	63 (19.6%)	90 (28%)	

Table7. Clinical significance analysis of PGAM1 in BLCA

Characteristics	Low expression of PGAM1	High expression of PGAM1	P value
n	206	206	
Pathologic T stage, n (%)			0.441
T1&T2	67 (17.7%)	56 (14.8%)	
T3	93 (24.6%)	103 (27.2%)	
T4	28 (7.4%)	31 (8.2%)	
Pathologic N stage, n (%)			0.809
N0	114 (31%)	124 (33.7%)	
N1	21 (5.7%)	25 (6.8%)	
N2&N3	43 (11.7%)	41 (11.1%)	
Pathologic M stage, n (%)			0.907
M0	106 (50%)	95 (44.8%)	
M1	6 (2.8%)	5 (2.4%)	
Pathologic stage, n (%)			0.197
Stage I	3 (0.7%)	1 (0.2%)	
Stage II	73 (17.8%)	56 (13.7%)	
Stage III	64 (15.6%)	78 (19%)	
Stage IV	66 (16.1%)	69 (16.8%)	
Primary therapy outcome, n (%)			**0.022**
PD	24 (6.8%)	46 (13%)	
SD	17 (4.8%)	13 (3.7%)	
PR	11 (3.1%)	11 (3.1%)	
CR	128 (36.1%)	105 (29.6%)	
